# Exercise training improves mitochondrial respiration and is associated with an altered intramuscular phospholipid signature in women with obesity

**DOI:** 10.1007/s00125-021-05430-6

**Published:** 2021-03-26

**Authors:** Amy E. Mendham, Julia H. Goedecke, Yingxu Zeng, Steen Larsen, Cindy George, Jon Hauksson, Melony C. Fortuin-de Smidt, Alexander V. Chibalin, Tommy Olsson, Elin Chorell

**Affiliations:** 1 MRC/Wits Developmental Pathways for Health Research Unit, Faculty of Health Sciences, University of the Witwatersrand, Johannesburg, South Africa; 2 Division of Exercise Science and Sports Medicine, Department of Human Biology, University of Cape Town, Cape Town, South Africa; 3 Non-communicable Diseases Research Unit, South African Medical Research Council, Cape Town, South Africa; 4 Hainan Tropical Ocean University, Sanya, Hainan China; 5 Department of Public Health and Clinical Medicine, Umeå University, Umeå, Sweden; 6 Center for Healthy Aging, Department of Biomedical Sciences, Copenhagen University, Copenhagen, Denmark; 7 Clinical Research Centre, Medical University of Bialystok, Bialystok, Poland; 8 Department of Radiation Sciences, Radiation Physics and Biomedical Engineering, Umeå University, Umeå, Sweden; 9 Department of Molecular Medicine and Surgery, Karolinska Institutet, Stockholm, Sweden

**Keywords:** Acylcarnitines, Aerobic and resistance training, Cardiolipins, Cardiorespiratory fitness, Ectopic fat, Mitochondrial biogenesis, Obesity, Phospholipid hydrolysis, Sphingomyelin, Triacylglycerol

## Abstract

**Aims/hypothesis:**

We sought to determine putative relationships among improved mitochondrial respiration, insulin sensitivity and altered skeletal muscle lipids and metabolite signature in response to combined aerobic and resistance training in women with obesity.

**Methods:**

This study reports a secondary analysis of a randomised controlled trial including additional measures of mitochondrial respiration, skeletal muscle lipidomics, metabolomics and protein content. Women with obesity were randomised into 12 weeks of combined aerobic and resistance exercise training (*n* = 20) or control (*n* = 15) groups. Pre- and post-intervention testing included peak oxygen consumption, whole-body insulin sensitivity (intravenous glucose tolerance test), skeletal muscle mitochondrial respiration (high-resolution respirometry), lipidomics and metabolomics (mass spectrometry) and lipid content (magnetic resonance imaging and spectroscopy). Proteins involved in glucose transport (i.e. GLUT4) and lipid turnover (i.e. sphingomyelin synthase 1 and 2) were assessed by western blotting.

**Results:**

The original randomised controlled trial showed that exercise training increased insulin sensitivity (median [IQR]; 3.4 [2.0–4.6] to 3.6 [2.4–6.2] x10^−5^ pmol l^−1^ min^−1^), peak oxygen consumption (mean ± SD; 24.9 ± 2.4 to 27.6 ± 3.4 ml kg^−1^ min^−1^), and decreased body weight (84.1 ± 8.7 to 83.3 ± 9.7 kg), with an increase in weight (pre intervention, 87.8± 10.9 to post intervention 88.8 ± 11.0 kg) in the control group (interaction *p* < 0.05). The current study shows an increase in mitochondrial respiration and content in response to exercise training (interaction *p* < 0.05). The metabolite and lipid signature at baseline were significantly associated with mitochondrial respiratory capacity (*p* < 0.05) but were not associated with whole-body insulin sensitivity or GLUT4 protein content. Exercise training significantly altered the skeletal muscle lipid profile, increasing specific diacylglycerol(32:2) and ceramide(d18:1/24:0) levels, without changes in other intermediates or total content of diacylglycerol and ceramide. The total content of cardiolipin, phosphatidylcholine (PC) and phosphatidylethanolamine (PE) increased with exercise training with a decrease in the PC:PE ratios containing 22:5 and 20:4 fatty acids. These changes were associated with content-driven increases in mitochondrial respiration (*p* < 0.05), but not with the increase in whole-body insulin sensitivity or GLUT4 protein content. Exercise training increased sphingomyelin synthase 1 (*p* < 0.05), with no change in plasma-membrane-located sphingomyelin synthase 2.

**Conclusions/interpretation:**

The major findings of our study were that exercise training altered specific intramuscular lipid intermediates, associated with content-driven increases in mitochondrial respiration but not whole-body insulin sensitivity. This highlights the benefits of exercise training and presents putative target pathways for preventing lipotoxicity in skeletal muscle, which is typically associated with the development of type 2 diabetes.

**Graphical abstract:**

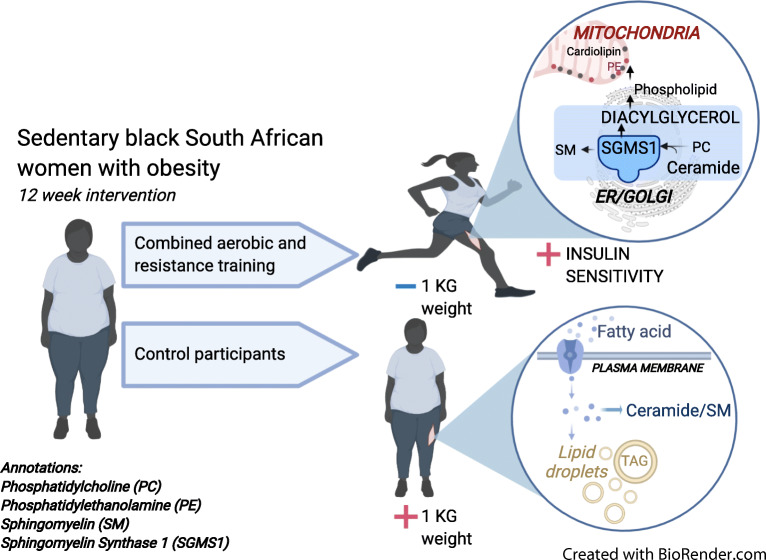

**Supplementary Information:**

The online version contains peer-reviewed but unedited supplementary material available at 10.1007/s00125-021-05430-6.

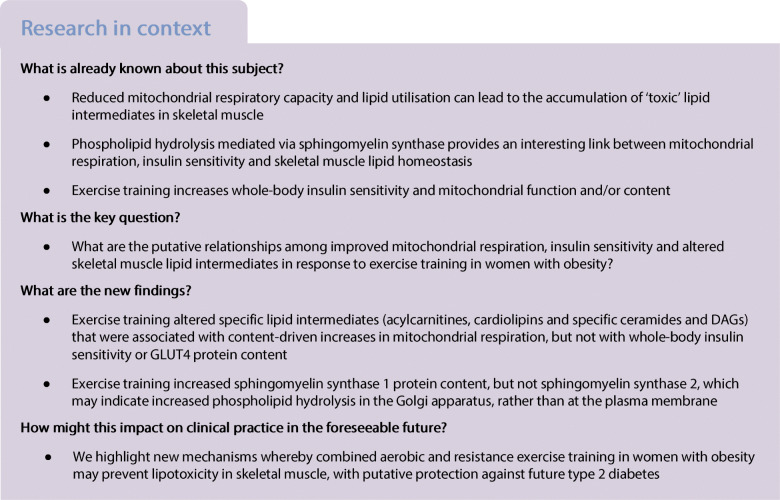



## Introduction

Low levels of physical activity and cardiorespiratory fitness are two components contributing to the increasing rates of obesity [1]. Obesity is closely associated with the redistribution of fatty acids to ectopic sites, such as skeletal muscle, where a reduced mitochondrial content and function leads to accumulation of fatty acids and their potentially harmful by-products [2–4]. Specifically, reduced mitochondrial respiratory capacity with reduced lipid utilisation can lead to the accumulation of ‘toxic’ lipid intermediates in skeletal muscle, which may be key to the development of insulin resistance and type 2 diabetes [2, 4, 5]. Nevertheless, the mechanisms of muscle lipotoxicity remain unclear and are masked by contradictory results relating to the chemical diversity and sub-cellular localisation of lipids [6–11]. In this, putative influences of mitochondrial biogenesis and whole-body insulin sensitivity on skeletal muscle lipid intermediates may play important roles [8, 12–14].

Lipid intermediates such as diacylglycerols (DAGs) and ceramides are considered ‘lipotoxic’ and contribute to muscle insulin resistance [7, 8]. Mechanistically, both DAGs and ceramides may block important enzymatic pathways that affect the muscle’s responsiveness to insulin [6, 15]. Notably, DAGs and ceramides are versatile in their chemical structure, and consist of numerous variants of fatty acyl combinations that influence their biological activity and their insulin-desensitising effects [11]. In fact, total DAG levels in skeletal muscle are comparable between individuals with type 2 diabetes and insulin sensitive athletes [11]. Similar to DAGs, ceramides’ biological activity and ability to induce organ/organelle dysfunction lies in their localisation and acyl composition [10].

An important regulator of bioactive DAG and ceramide tissue levels is phospholipid hydrolysis [16]. Phospholipid hydrolysis is partly regulated through the action of sphingomyelin synthases, which transfer a phosphocholine group from phosphatidylcholine (PC) into ceramides, with the release of sphingomyelin and DAG [17]. Two sphingomyelin synthase isoforms are expressed in muscle cells, with sphingomyelin synthase 1 (SGMS1) predominately localised in the Golgi apparatus and sphingomyelin synthase 2 (SGMS2) at the plasma membrane. Notably, SGMS1-null mice present with insulin secretion deficiencies and mitochondrial dysfunction [18], whereas SGMS2 knockout mice are protected against high-fat diet-induced obesity and insulin resistance [19]. Accordingly, SGMS1 is the dominant sphingomyelin synthase at the Golgi apparatus and produces DAGs that pool in the endoplasmic reticulum (ER)/Golgi network to produce phospholipid intermediates [20]. Research in mammals suggests that phospholipids in the ER/Golgi network are transported to the mitochondria for the synthesis of cardiolipins and phosphatidylethanolamine (PE) [21], which can have direct repercussions on mitochondrial function and content [22, 23]. Altogether, phospholipid hydrolysis, mediated via sphingomyelin synthase, provides an interesting link between mitochondrial function, insulin sensitivity and skeletal muscle lipid homeostasis.

Exercise training improves whole-body and skeletal muscle insulin sensitivity, which is further coupled with mitochondrial biogenesis [24–26]. Changes in specific intermediates of phospholipids, DAGs and ceramides may therefore provide further insight into mechanisms of improved mitochondrial function and insulin sensitivity in response to exercise training. We sought to determine putative relationships among mitochondrial function, insulin sensitivity and altered skeletal muscle lipids and metabolites in response to an exercise intervention in black South African women with obesity. Young black South African women were selected for this study due to their high risk of excessive weight gain and associated decline in insulin sensitivity [27]. We hypothesised that combined aerobic and resistance exercise training would induce improvements in mitochondrial respiration and insulin sensitivity associated with altered skeletal muscle lipid signature, including changes in sphingomyelin synthases, phospholipids, DAGs and ceramides.

## Methods

### Study design

This study is a secondary analysis from a randomised control trial, for which information on recruitment, retention, methods and sample size determination have previously been reported [28]. Primary and secondary endpoints (insulin sensitivity, peak oxygen consumption [$\dot{V}{\mathrm{O}}_{2\mathrm{peak}}$] and body composition) from the original randomised control trial have been previously reported [29] and additional measures of mitochondrial respiration, skeletal muscle lipidomics, metabolomics and protein content not determined in the original study were analysed by the authors of this paper. All participants were recruited from a low socioeconomic urban area in Cape Town between July 2015 and December 2016. One hundred and eighteen women were screened and assessed for eligibility, of whom 45 obese sedentary black South African women were eligible and block (2–4 participants) randomised into control (no exercise, *n* = 22) or experimental (exercise, *n* = 23) groups. Block randomisation and group allocation were performed by the project manager after participants completed pre-intervention testing to ensure that investigators performing the testing were blinded to group allocation. Ten participants dropped out or were lost to follow-up in the exercise (*n* = 3) and control groups (*n* = 7). The study was approved by the Human Research Ethics Committee at the University of Cape Town (HREC REF: 054/2015) and is a secondary analysis of a registered trial in the Pan African Clinical Trial Registry (No. 201711002789113; https://pactr.samrc.ac.za/). The study was performed in accordance with the principles of the Declaration of Helsinki as revised in 2008, ICH Good Clinical Practice (GCP), and the laws of South Africa. Participants provided written informed consent before participation.

### Participants

Participant recruitment ensured the following inclusion criteria: black South African women (based on the *isiXhosa* ancestry of both parents), 20–35 years of age, BMI of 30–40 kg/m^2^, weight stable (weight not changed more than 5 kg over the past 6 months), sedentary (completing <1 session of exercise <20 min per week within the last 12 months). Different hormonal contraceptives have differential effects on fasting glucose and insulin levels [30]. We standardised the contraceptive method as the most commonly prescribed in a South African cohort (depot medroxyprogesterone acetate, 400 mg) [31] for a minimum of 2 months. Exclusion criteria included known metabolic or inflammatory diseases, hypertension (≥140/90 mmHg; Omron 711, Omron Health Care, Hamburg, Germany), diabetes (random plasma glucose concentration of >11.1 mmol/l, and/or HbA_1c_ [liquid chromatography, D-10 Hemoglobin Testing System, BIO-RAD, Johannesburg, South Africa] result >48 mmol/mol [>6.5%]), HIV positive (rapid anti-HIV [1&2]) test, Advanced quality, InTec Product, Xiamen, China), or anaemia (haemoglobin <120 g/l), taking medications, smoking, orthopaedic or medical problems that may prevent exercise participation, and surgical procedures within the last 6 months.

### 12 week intervention

**Fig. 1:**
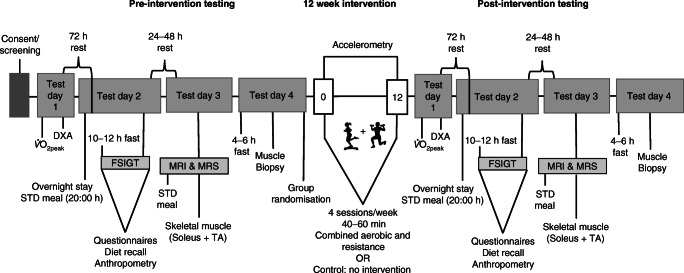
Schematic overview of testing timeline and procedures. Abbreviations: FSIGT, frequently sampled intravenous glucose tolerance test; STD, standard; TA, tibialis anterior. This figure is adapted from Goedecke et al [28], licensed under a Creative Commons Attribution License (https://creativecommons.org/licenses/by/4.0/)

A schematic overview of testing timeline, procedures and intervention has been described previously [28] and is summarised in Fig. 1. The exercise intervention consisted of 12 weeks of supervised aerobic and resistance training at a moderate-vigorous intensity for 40–60 min, 4 days/week by a trained facilitator, which included a 50% time split between the two modalities [29]. Aerobic exercises included dance, running, skipping and stepping at a moderate-vigorous intensity (75–80% peak heart rate [HR_peak_]). Resistance exercises included body weight exercise that progressed to the use of equipment (i.e. bands and free weights). These exercises included squats, lunges, bicep curls, push-ups and shoulder press with a prescribed intensity of 60–70% HR_peak_. Heart rate monitors (Polar A300, Kempele, Finland) were worn to ensure the prescribed exercise intensity was maintained. Both groups were instructed to maintain their normal dietary intake and physical activity patterns, which were objectively quantified at baseline and weeks 4, 8 and 12. Following post-intervention testing, the control participants were provided with the opportunity to participate in the 12 week supervised exercise programme.

### Pre- and post-intervention testing

#### Body composition assessment

Basic anthropometry measurements included weight, as well as height, waist circumference (level of umbilicus) and hip circumference (largest protrusion of the buttocks), measured to the nearest 0.1 cm. Whole-body composition, including subtotal (excluding the head) fat mass and fat-free soft tissue mass, were measured by dual-energy x-ray absorptiometry (DXA; Discovery-W, software version 12.7.3.7; Hologic, Bedford, MA, USA) according to standard procedures. Regional body fat distribution, including gynoid and android fat mass, was characterised as previously described [32].

#### Cardiorespiratory fitness

A walking, treadmill-based (C, Quasar LE500CE, HP Cosmos, Nussdorf-Traunstein, Germany) graded exercise test until volitional exhaustion was conducted. $\dot{V}{\mathrm{O}}_{2\mathrm{peak}}$ and HR_peak_ (Polar A300, Kempele, Finland) was reported. Pulmonary gas exchange was measured by determining O_2_ and CO_2_ concentrations and ventilation to calculate $\dot{V}{\mathrm{O}}_2$ using a metabolic gas analysis system (CPET, Cosmed, Rome, Italy). A two-point calibration was conducted prior to each test, as previously described [28].

#### Intravenous glucose tolerance test

Participants stayed overnight at the laboratory and were provided a standardised meal (energy 2456 kJ, 21 g protein, 49 g carbohydrate and 32 g fat) at 20:00 h followed by an overnight fast (10–12 h). At post intervention, this testing was completed 72 h following the last exercise training session (Fig. 1). Baseline samples were collected at −5 and −1 min before a bolus of glucose (50% dextrose; 11.4 g/m^2^ body surface area) was infused intravenously over 60 s beginning at time 0. At 20 min, human insulin (0.02 U/kg; NovoRapid, Novo Nordisk, Kalundborg, Denmark) was infused over 5 min at a constant rate (HK400 Hawkmed Syringe Pump, Shenzhen Hawk Medical Instrument, Shenzhen, China) and samples were collected up to 240 min for the subsequent analysis of serum insulin (IMMULITE 1000 immunoassay system, Siemens Healthcare, Midrand, South Africa) and plasma glucose (Randox, Gauteng, South Africa) concentrations. Bergman’s minimal model of glucose kinetics was used to calculate the insulin sensitivity index (S_I_) [33].

#### Ectopic lipid content

After a standardised meal and drink (energy 2553 kJ, protein 21 g, carbohydrate 83 g, fat 22 g), MRI and magnetic resonance spectroscopy (MRS) were used to determine skeletal muscle (soleus, tibialis anterior) fat content, and intra-myocellular (IMCL) and extra-myocellular lipid content (EMCL), respectively. These investigations were completed using a 3 Tesla whole-body human MRI scanner (MAGNETOM Skyra, Siemens Medical Solutions, Erlangen, Germany) as described previously [3, 29].

#### Skeletal muscle biopsies

Muscle samples were collected after a 4–6 h fast and at least 48–72 h after the last exercise session. After local anaesthesia (2% lidocaine hydrochloride, Intramed, Port Elizabeth, South Africa), a skeletal muscle biopsy was collected from *m. vastus laterali*s using a 5 mm Bergstrom needle. A subsample of tissue was placed in ice-cold BIOPS relaxation buffer [34] for immediate analysis of mitochondrial respiration. The remaining samples were frozen immediately in liquid nitrogen and stored at −80°C for the analysis of metabolomics, lipidomics and proteins involved in mitochondrial respiration, phospholipid metabolism, glucose transport and insulin signalling.

#### Muscle preparation and western blotting

Details of muscle preparation and western blotting are reported in the electronic supplementary material (ESM) Methods. The following primary antibodies were used for protein quantification and diluted according to the manufacturer’s instructions; mitochondrial oxidative phosphorylation (OXPHOS) cocktail (Mitosciences, Eugene, OR, USA, #MS601), hormone sensitive lipase (HSL; Cell Signaling Technology, Danvers, MA, USA, #CS4107), hydroxyacyl-CoA dehydrogenase (HADHSC; Abcam, Cambridge, MA, USA, #ab154088), mammalian target of rapamycin (mTOR; Cell Signaling, Danvers, MA, USA, #CS2983), acetyl-CoA carboxylase (ACC; Cell Signaling, Danvers, MA, USA, #CS3676), phospholipase A2γ (iPLA2γ; Abcam, #ab154233), SGMS1 (Abcam, #ab135365), SGMS2 (Abcam, #ab87214), IRS1 (Millipore, Darmstadt, Germany, #06-248), citrate synthase (Abcam, #ab96600), adipose triglyceride lipase (ATGL; Abcam, #ab109251), glycerol-3-phosphate acyltransferase 1 (GPAT1; Abcam, #ab69990), lysophosphatidylcholine acyltransferase (LPCAT)3 (Abbexa, Cambridge, UK, #abx104290), GLUT4 (Millipore, #07-1404) and PPARG coactivator 1α (PGC-1α; Cell Signaling, #CS2178). Based on antibody manufacturer’s instructions, the lower band was quantified (band predicted at 42 kDa) for SGMS2 and the top band was quantified (band predicted at 94 kDa) for GPAT1.

#### Mitochondrial respiratory capacity

Measures of mitochondrial respiration were performed using high-resolution respirometry (Oxygraph-2k; Oroboros, Innsbruck, Austria). The multiple SUIT protocol included [25, 35]: (1) the addition of malate (2 mmol/l) and octanoyl-carnitine (0.2 mmol/l) represents lipid-induced leak respiration through the electron transferring flavoprotein (ETF) in the absence of adenylates (Leak^ETF^); (2) lipid OXPHOS capacity was induced with the addition of ADP (5 mmol/l) (ETF^p^); (3) state 3 respiration capacity (complex I) with the addition of pyruvate (5 mmol/l) and glutamate (10 mmol/l); (4) maximal state 3 respiration (complex I + II) OXPHOS capacity (succinate, 10 mmol/l); (5) state 4o respiration (oligomycin-induced leak respiration [Leak^Oly^]), through inhibition of ATP synthase using oligomycin (2.5 μmol/l); (6) electron transport system (ETS) capacity with the titration of carbonyl cyanide *m*-chlorophenyl hydrazone (0.5 μmol/l titration steps); (7) the inhibition of complex I with the addition of rotenone (0.5 μmol/l); (8) the inhibition of complex III with the addition of antimycin A (2.5 μmol/l). All respiration data are reported relative to mass in wet weight (pmol s^−1^ [mg w.w.]^−1^) and citrate synthase protein content as a marker of mitochondrial content (pmol s^−1^ [mg w.w.]^−1^/citrate synthase protein content [arbitrary units (AU)]). Citrate synthase protein content was divided into mass-specific respiration to calculate content-specific mitochondrial respiration, which is termed intrinsic mitochondrial respiration. See ESM Methods for further detail.

#### Metabolomic and lipidomic analyses

Multi-platform metabolomic and lipidomic analyses were performed on skeletal muscle samples, including gas chromatography time-of-flight mass spectrometry (GC-TOF/MS) and liquid chromatography time-of-flight mass spectrometry (LC-TOF/MS, operating in positive and negative ion modes). A complete description of metabolomics and lipidomics mass spectrometry methods are described in ESM Methods. Sample preparation procedures have been described previously [36, 37]. All lipids were annotated according to standard lipid nomenclature set by the Lipid Maps Lipidomics Gateway (lipidmaps.org), i.e. lipid class, total number of carbons in the attached fatty acids and total number of double bonds. Further information on lipid-specific fatty acid composition is provided for cases where a clear mass spectra fragmentation pattern was obtained, i.e. phosphatidylcholine(40:5) contains C18:0 and C22:5 fatty acids and was thus denoted as phosphatidylcholine(18:0/22:5). Notably, no differentiation could be made between isomers such as *sn*-1 and *sn*-2 positions of fatty acids. Of note, due to the high content of high-abundant lipids such as triacylglycerols (TAGs) and phospholipids in the skeletal muscle samples, a number of low-abundant DAG species were not possible to quantify. Further details on metabolomic and lipidomic analyses of skeletal muscle extracts, including relative abundance of detected lipid classes (ESM Fig. 1 and ESM Table 1) and data processing methods, are shown in ESM Methods.

#### Physical activity and dietary intake

Physical activity was measured using accelerometery (ActiGraph GTX3+, ActiGraph, Pensacola, FL, USA), at baseline and at week 12. The ActiGraph was worn on the hip for 24 h a day over a 7 day period. Total habitual time physical activity (≥100 cpm) and sedentary time (≤100 cpm) are reported on all days in the control group and non-training days in the exercise group (ActiLife software; Version 6, Pensacola, FL, USA). At the same time-points dietary intake was estimated using a 24 h recall and a 3 day dietary record, including 2 weekdays and 1 weekend day. Nutrient intake was calculated using the South African Food Composition Database System (SAFOOD, the South African Food Composition Database, South African Medical Research Council, Cape Town, South Africa).

### Statistical analyses

Data relating to mitochondrial respiration, protein expression, S_I_, body composition and muscle fat content was analysed using IBM SPSS statistics (Version 25, Statistical Package for the Social Sciences, Chicago, IL, USA). Incomplete data on several participants occurred and was treated as missing data for the analysis. Final participant numbers for baseline analysis (*n* = 40) represents pooled groups at pre-intervention. Analysis of the intervention report *n* = 20 for exercise and *n* = 15 for control groups unless otherwise specified in figure captions and table legends. Normally distributed data are expressed as mean ± SD and non-normally distributed data are expressed as median (IQR) and transformed prior to analysis. Response to the intervention was analysed using repeated measures ANOVA, with Fisher’s least significant difference post hoc test. Missing data within each variable excluded the respective participant/s. Statistical significance (α) was set at *p* < 0.05.

Multivariate analysis was used for all lipidomic and metabolomic data by MATLAB R2016a (The MathWorks, Natick, MA, USA) and SIMCA 16 software (Sartorius, Umetrics, Umeå, Sweden). Multivariate analyses included principal component analysis to first inspect data in terms of groupings, outliers and general trends. The intervention-specific skeletal muscle metabolite and lipid signatures were calculated in orthogonal partial least squares-effect projections (OPLS-EP) models [38]. OPLS-EP is a variant of OPLS and highly suitable for investigating the intervention-related response since it is calculated from each individual’s delta values, where each participant’s pre-intervention skeletal muscle metabolite measure is subtracted from its post-intervention measure. By using this approach, we can evaluate the intervention-specific effect on the metabolites and lipids with minimal influence from instrumental drift and inter-individual variation. In addition, OPLS analysis was applied to explore associations between changes in mitochondrial respiratory capacity (complex I + II-linked mitochondrial respiration) and insulin sensitivity with changes in skeletal muscle lipids and metabolites. Separate OPLS models were calculated for control and exercise training groups. Prior to multivariate analyses, all included metabolites and lipids were scaled to unit variance to prevent low-abundant compounds being masked by high-abundant ones. All discussed metabolite profiles, if not stated, are significant based on the latent significant criteria using a 95% confidence level [39] and all models were validated based on the ANOVA of the cross-validated OPLS scores (CV-ANOVA) [40]. Jack-knifing-based confidence intervals were calculated from cross-validation to display significance of unique variables (metabolites) in multivariate models [41].

## Results

### Compliance and participant characteristics in response to the 12 week intervention

Of the 48 exercise sessions, participants attended 79 ± 13% (range 52–100%) at a mean intensity of 79.7 ± 4.0% (range 71–85%) HR_peak_ [42]. There was no change in total habitual physical activity or sedentary time in response to the intervention (Table 1). Daily energy intake and macronutrient consumption throughout the intervention have been previously reported [43]. Changes in body composition, $\dot{V}{\mathrm{O}}_{2\mathrm{peak}}$, S_I_ and skeletal muscle lipid content in response to the intervention are presented in Table 1, with several variables previously published [29]. The exercise training group showed increases in $\dot{V}{\mathrm{O}}_{2\mathrm{peak}}$ and S_I_ (*p* < 0.05), with no changes in the control group. Body weight decreased in the exercise group, and increased in the control group (interaction effect, *p* = 0.003).

**Table 1:** Body composition, S_I_ and skeletal muscle lipid content at baseline and in response to the 12 week intervention

Variable	Exercise		Control		Group	Time	Interaction
	Pre	Post	Pre	Post	*p* value	*p* value	*p* value
Age (years)	23 ± 3	–	24 ± 4	–			
BMI (kg/m^2^)	34.1 ± 2.8	33.8 ± 3.1*	33.4 ± 2.7	33.8 ± 2.8*	0.430	0.038	0.003
Weight (kg)	84.1 ± 8.7	83.3 ± 9.7*	87.8± 10.9	88.8 ± 11.0*	0.267	0.030	0.003
$\dot{V}{\mathrm{O}}_{2\mathrm{peak}}$ (ml kg^−1^ min^−1^)	24.9 ± 2.4	27.6 ± 3.4*	23.9 ± 2.8	22.9 ± 2.6	0.291	0.195	<0.001
$\dot{V}{\mathrm{O}}_{2\mathrm{peak}}$ (ml/min)	2078 ± 211	2278 ± 231*	2099 ± 282	2032 ± 196	0.447	0.144	<0.001
S_I_ × 10^−5^ (pmol l^−1^ min^−1^)	3.4 (2.0–4.6)	3.6 (2.4–6.2)*	4.2 (2.1–6.8)	3.4 (2.4–6.2)	0.094	0.711	0.037
Physical activity							
Total physical activity (min/day)	352.6 ± 50.8	401.9 ± 61.4	398.8 ± 97.2	378.6 ± 104.6	0.561	0.459	0.081
Total sedentary (min/day)	515.6 ± 59.8	478.3 ± 86.5	467.6 ± 69.8	509.4 ± 102.7	0.910	0.677	0.054
DXA							
Fat mass (%)	49.9 (48.5–51.6)	49.9 (48.3–51.0)	49.8 (46.7–52.7)	50.9 (47.7–52.9)	0.981	0.480	0.471
FFSTM (kg)	37.1 (33.5–39.5)	37.1 (33.7–39.9)	37.7 (34.6–40.8)	38.2 (35.2–40.9)	0.293	0.223	0.324
MRI and MRS							
Soleus IMCL (%)	2.95 (2.57–4.39)	2.73 (2.36–3.63)	2.83 (2.23–3.89)	2.57 (1.87–4.44)	0.283	0.353	0.893
Soleus EMCL (%)	4.49 (3.24–5.72)	3.64 (2.83–5.02)	4.35 (3.61–7.35)	5.38 (3.68–9.00)	0.059	0.485	0.387
Soleus fat (%)	10.4 (7.4–12.7)	9.9 (8.4–11.2)	9.7 (8.5–10.8)	9.5 (8.6–11.3)	0.478	0.682	0.522
Tibialis anterior IMCL (%)	0.44 (0.24–0.64)	0.47 (0.30–0.56)	0.40 (0.29–0.47)	0.38 (0.19–0.46)	0.088	0.785	0.320
Tibialis anterior EMCL (%)	1.54 (1.43–3.72)	2.14 (1.89–3.51)	2.93 (1.97–5.14)	2.87 (1.67–5.34)	0.150	0.865	0.588
Tibialis anterior fat (%)	5.0 (2.9–6.3)	4.2 (3.3–5.4)	3.4 (2.7–3.9)	4.0 (32.7–4.7)	0.674	0.979	0.554

Data reported as mean ± SD for normally distributed variables and as median (IQR) for skewed variables

Repeated measures ANOVA identified main effect of time (pre and post), group (exercise and control), and interaction (group × time) in exercise and control groups

Significant change within the group, *p < 0.05. MRI and MRS data represents *n* = 13 in control and *n* = 20 in exercise groups

FFSTM, fat-free soft tissue mass

### Mitochondrial respiration in response to the 12 week intervention

**Fig. 2:**
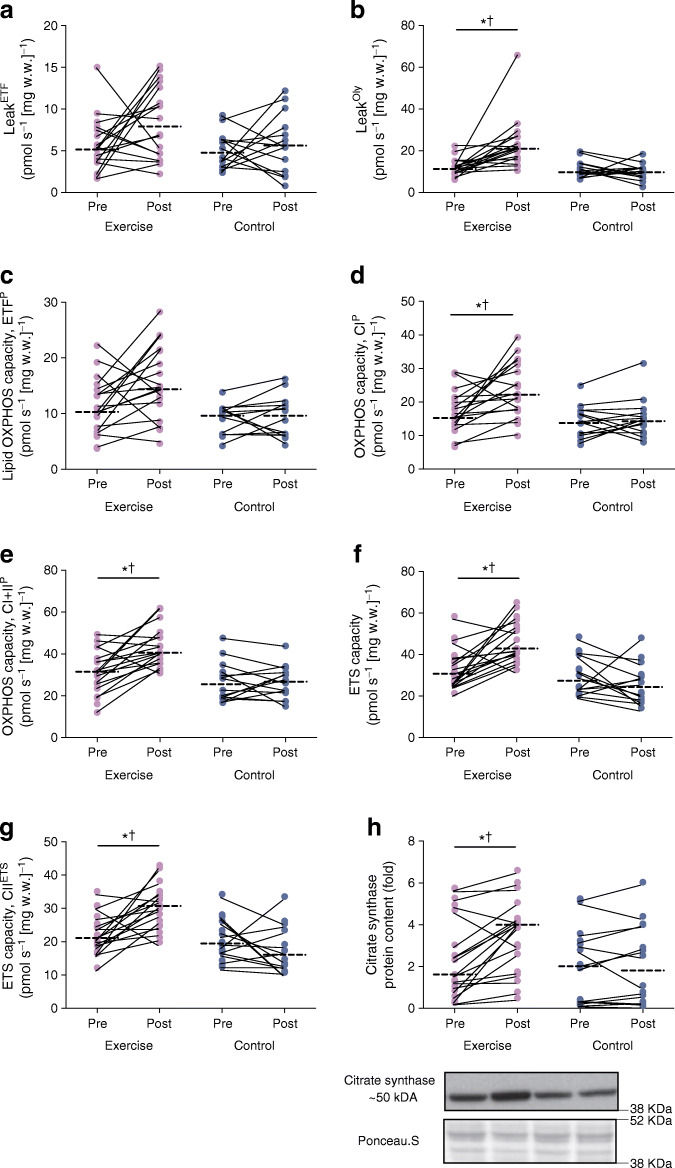
Mitochondrial respiration across all respiratory states (pmol s^−1^ [mg w.w.]^−1^). (**a**) Lipid-induced leak respiration through the ETF in the absence of adenylates (Leak^ETF^). (**b**) State 4o respiration, oligomycin-induced leak respiration (Leak^Oly^) through inhibition of ATP synthase. (**c**) Lipid OXPHOS capacity (ETF^p^). (**d**) State 3 OXPHOS capacity specific to ETF and complex I (CI^p^). (**e**) Maximal state 3 OXPHOS capacity (CI+II^p^). (**f**) ETS capacity. (**g**) ETS capacity through complex II (CII^ETS^). (**h**) Citrate synthase protein expression with representative blots in response to the 12 week intervention. Repeated measures ANOVA identified main effect of time (pre and post), group (exercise and control), and interaction (group × time) in exercise (*n* = 18) and control (*n* = 14) groups. The dashed line represents the median. Significant difference between pre and post intervention *p < 0.05. Significant group × time interaction ^†^*p* < 0.05

**Fig. 3:**
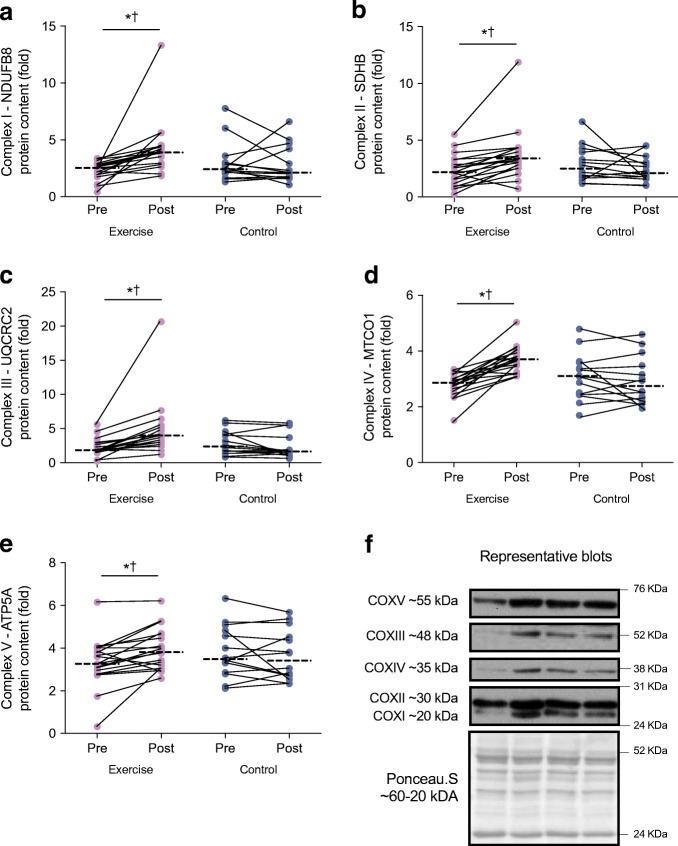
OXPHOS protein expression of (**a**–**e**) complex I–V protein expression in response to the 12 week intervention and (**f**) representative blots. Repeated measures ANOVA identified main effect of time (pre and post), group (exercise and control), and interaction (group × time) in exercise (*n* = 18) and control (*n* = 14) groups. Dashed line represents median. Significant difference between pre and post intervention *p < 0.05. Significant group × time interaction ^†^*p* < 0.05

Changes in absolute (mg w.w. adjusted) mitochondrial respiration and protein content of citrate synthase in response to the intervention are presented in Fig. 2. With the exception of Leak^ETF^ and ETF^p^, all other respiratory states and citrate synthase protein content increased in response to the exercise training (interaction *p* < 0.05), without changes in the control group. When adjusted for the increase in citrate synthase protein content as a marker of mitochondrial content, there were no changes in mitochondrial respiration across all respiratory states in the exercise and control groups (ESM Fig. 2). Protein content for all mitochondrial complexes (I-V) increased in response to exercise training (Fig. 3).

### Skeletal muscle metabolomics and lipidomics

**Fig. 4:**
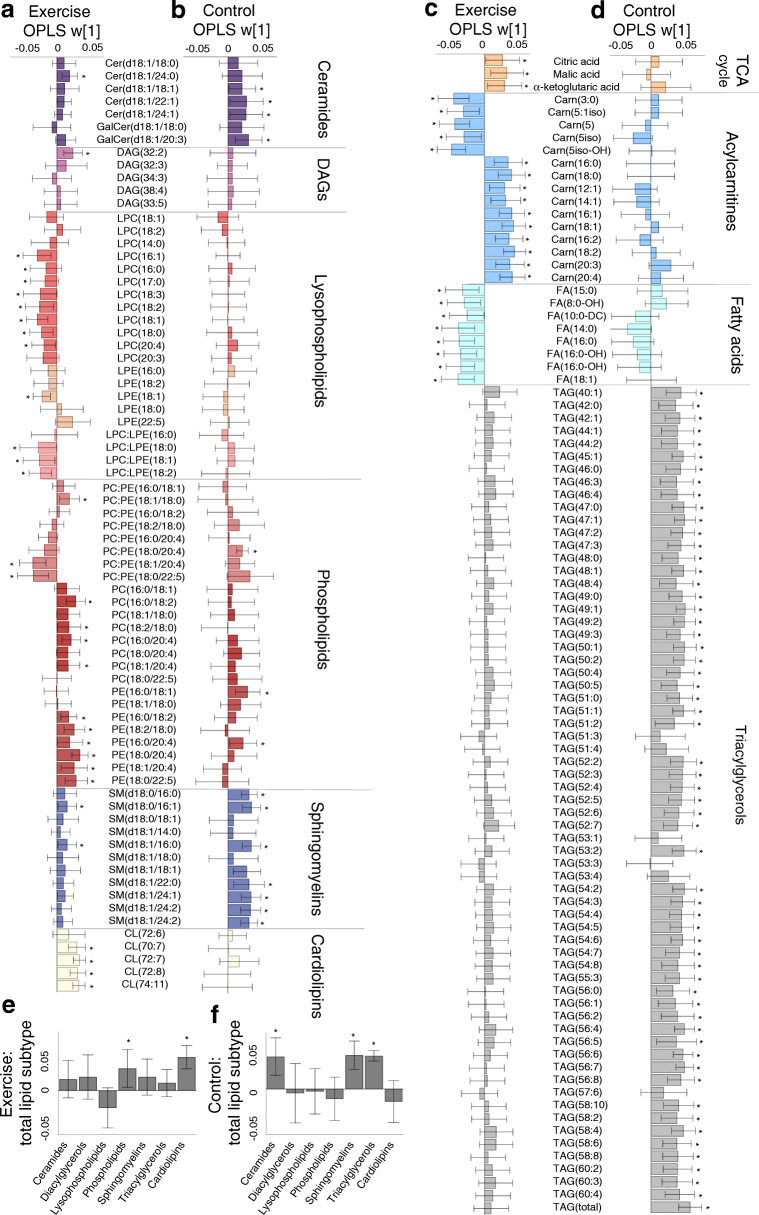
The intervention-specific multivariate response in skeletal muscle lipids and metabolites from 12 weeks of (**a**, **c**, **e,** OPLS-EP, CV-ANOVA *p* < 0.05) exercise training (*n* = 19) and (**b**, **d**, **f,** OPLS-EP, CV-ANOVA *p* < 0.05) control participants (*n* = 14). Metabolites/lipids that are significantly altered with exercise training are highlighted by an asterisk (*) using confidence intervals that indicate a 95% confidence level (jack-knifing statistics) and w[1] defines the calculated latent OPLS-EP variable. A complete table of all detected metabolites and lipids included in models can be found in ESM Table 1. Annotation of metabolites and lipids: cardiolipin (CL); carnitine (Carn); ceramides (Cer); diacylglycerol (DAG); fatty acid (FA); galactose (Gal); lysophosphatidylcholine (LPC); lysophosphatidylethanolamine (LPE); phosphatidylcholine (PC); phosphatidylethanolamine (PE); sphingomyelin (SM); triacylglycerol (TAG); tricarboxylic acid cycle (TCA)

All detected and annotated skeletal muscle metabolites and lipids are listed in ESM Table 1. The intervention resulted in significantly altered skeletal muscle metabolite and lipid profiles in the control and exercise groups (Fig. 4; OPLS-EP, CV-ANOVA *p* < 0.001). Exercise training increased total content of cardiolipins and phospholipids, with no changes in total DAG, ceramide and TAG (Fig. 4e); however, the increase in cardiolipins was no longer significant when adjusting for mitochondrial content (citrate synthase; data not shown). Subtypes of lipid intermediates showed an increase in ceramide(d18:1/24:0), DAG(32:2) and cardiolipins in response to exercise training. Exercise training increased PEs containing polyunsaturated fatty acyls more than PCs, which resulted in a decrease in respective PC:PE ratios (Fig. 4a), a process indicative of phospholipid remodelling and the generation of deacylated lysoforms [44]. We found that exercise increased the remodelling-linked lysophosphatidylethanolamine(22:5), whereas the non-remodelled LPE i.e. LPE(18:1) decreased. In contrast to LPEs, all lysophosphatidylcholines (LPCs) decreased in response to exercise training, with no change in the control group. Exercise training also increased skeletal muscle acylcarnitines linked to fatty acid mobilisation and availability of tricarboxylic acid cycle (TCA) intermediates and decreased catabolic intermediates of branched-chain amino acids (BCAAs; Fig. 4c).

The control group increased total TAG, ceramides and sphingomyelin (Fig. 4f) and increased remodelled PC:PE ratios with no change in their corresponding lysophospholipids (Fig. 4b). Acylcarnitines, fatty acids and TCA intermediates were not altered in controls (Fig. 4d).

### Skeletal muscle metabolite and lipid association with S_I_, GLUT4 and mitochondrial respiration

**Fig. 5:**
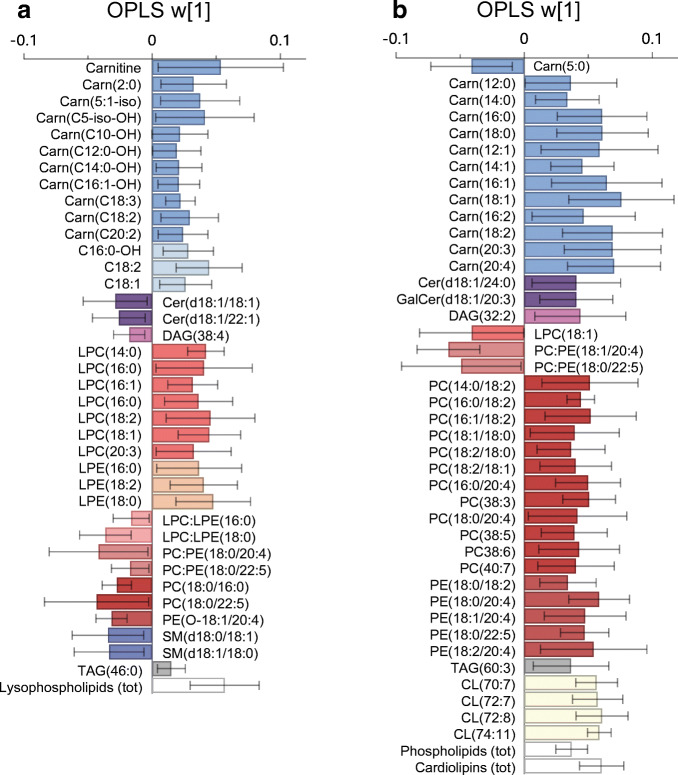
Multivariate association between (**a**) baseline skeletal muscle lipids and metabolites with mitochondrial respiratory capacity (*n* = 40 pooled exercise and control groups; OPLS, CV-ANOVA *p* = 0.001), and (**b**) change in response to exercise training in skeletal muscle lipids and metabolites with changes in mitochondrial respiratory capacity (*n* = 19; OPLS, CV-ANOVA *p* = 0.045). All shown metabolites are significant on a 95% confidence level and w[1] defines the latent OPLS variable. Annotation of metabolites and lipids: cardiolipin (CL); carnitine (Carn); ceramides (Cer); diacylglycerol (DAG); galactose (Gal); lysophosphatidylcholine (LPC); lysophosphatidylethanolamine (LPE); phosphatidylcholine (PC); phosphatidylethanolamine (PE); triacylglycerol (TAG); tot, total

The muscle metabolite and lipid signatures were not associated with S_I_ or GLUT4 protein content at baseline or in response to exercise training (OPLS CV-ANOVA *p* > 0.05). Instead, mitochondrial respiratory capacity (mg w.w.) was significantly associated with a metabolite and lipid signature at baseline (OPLS, CV-ANOVA *p* = 0.001, Fig. 5a) and in response to exercise training (OPLS, CV-ANOVA *p* = 0.045, Fig. 5b). No significant association was found between the changes in skeletal muscle lipids and metabolites with changes in mitochondrial respiratory capacity in the control group (OPLS, CV-ANOVA *p* > 0.05). At baseline, higher mitochondrial respiratory capacity was associated with higher total lysophospholipids and acylcarnitines, and lower DAG(38:4), ceramide(d18:1/18:1), ceramide(d18:1/22:1) and phospholipids. Increased total content of cardiolipins and phospholipids with exercise training were significantly associated with increased mitochondrial respiratory capacity (Fig. 5b). The increase in mitochondrial OXPHOS capacity was associated with a decrease in PC:PE ratios that contain fatty acids with more double bonds, and an increase in cardiolipins, DAG(32:2), ceramide(d18:1/24:0), and galactose-ceramide(d18:1/20:3). When adjusting mitochondrial respiratory capacity for mitochondrial content (citrate synthase), there were no significant associations with altered metabolite and lipid signatures (OPLS-EP, CV-ANOVA *p* > 0.05). Suggesting that the aforementioned relationships between mitochondrial respiratory capacity and lipid intermediates were driven by changes in mitochondrial content.

### Protein expression

**Fig. 6:**
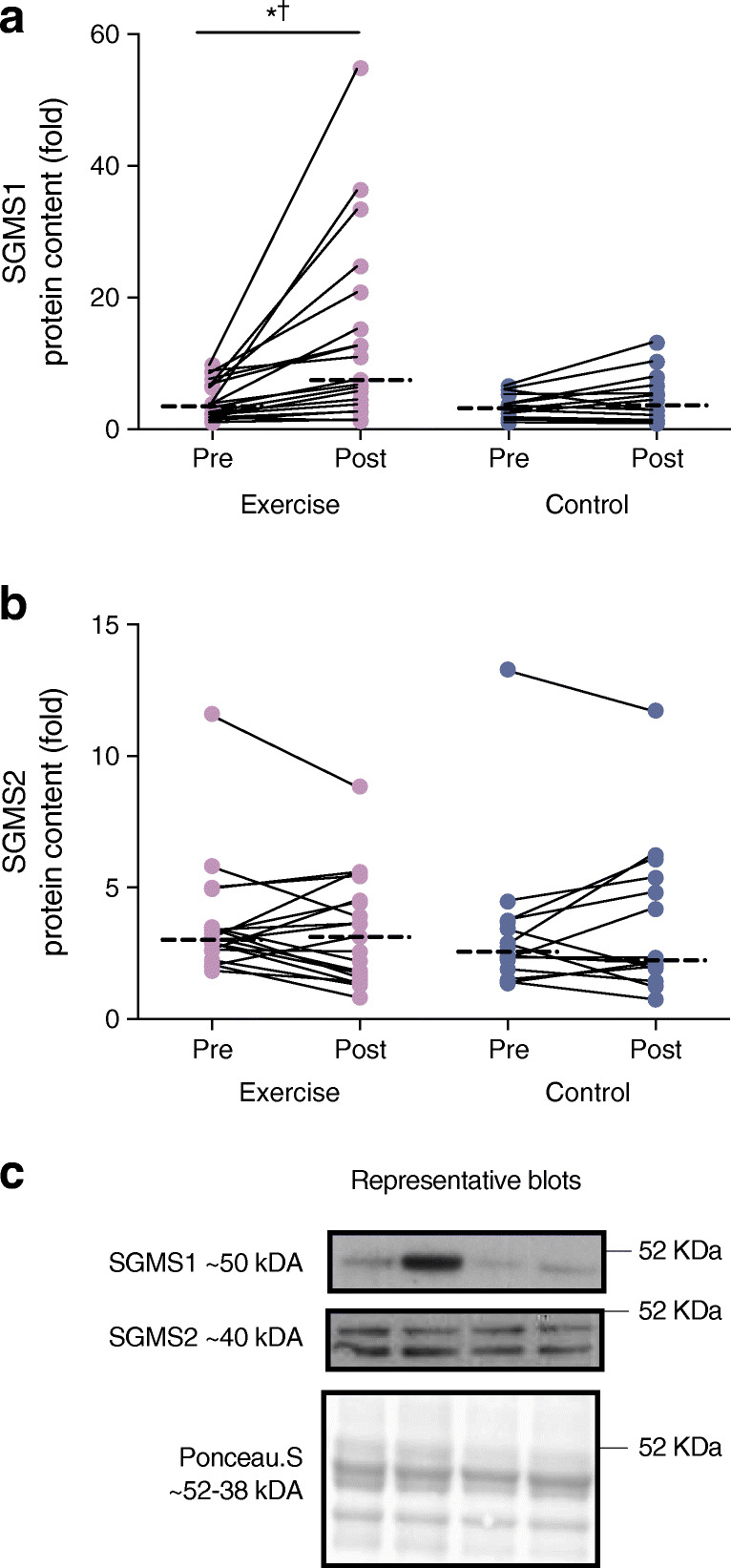
Protein expression of SGMS1 (**a**) and SGMS2 (**b**) in response to the 12 week intervention with (**c**) representative western blots. Repeated measures ANOVA was used to identify main effects of time (pre and post), group (exercise and control), and interaction (group × time) in exercise (*n* = 19) and control (*n* = 14) groups. The dashed line represents the median. Significant difference between pre and post intervention *p < 0.05. Significant group × time interaction ^†^*p* < 0.05

**Fig. 7:**
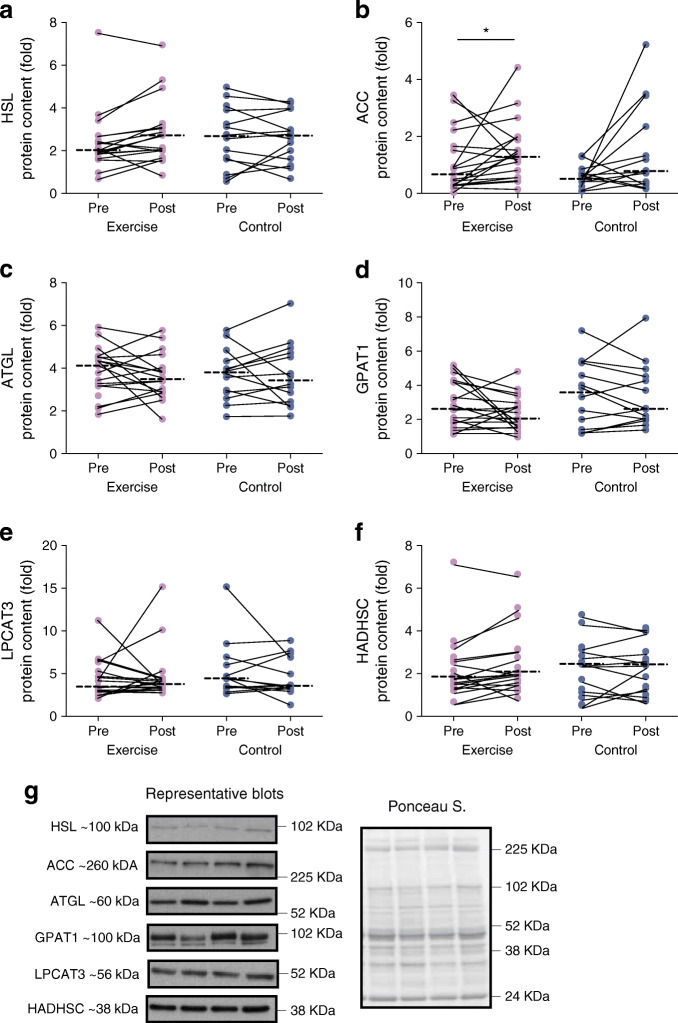
Protein expression for (**a**–**f**) markers of lipid metabolism and storage in response to the 12 week intervention with (**g**) representative blots. Repeated measures ANOVA was used to identify main effects of time (pre and post), group (exercise and control), and interaction (group × time) in exercise (*n* = 19) and control (*n* = 14) groups. The dashed line represents the median. Significant difference between pre and post intervention *p < 0.05

**Fig. 8:**
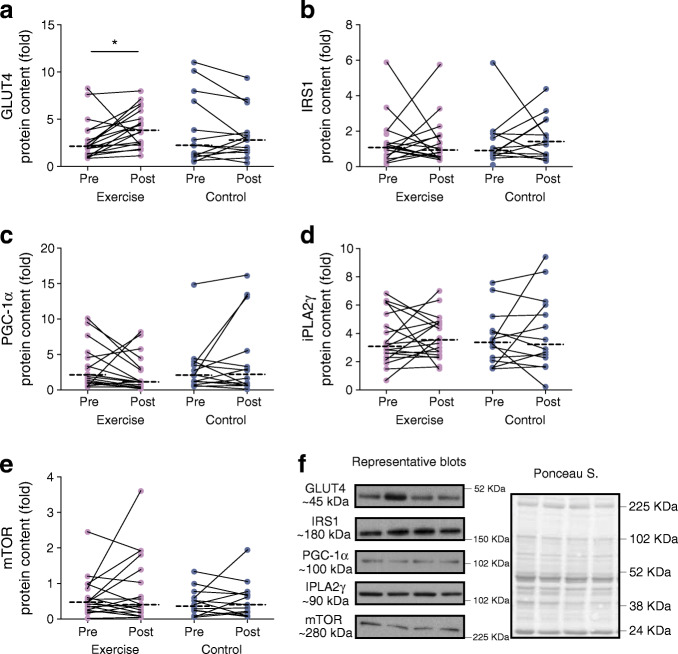
Protein expression for (**a**–**e**) markers of glucose metabolism in response to the 12 week intervention with (**f**) representative blots. Repeated measures ANOVA was used to identify main effects of time (pre and post), group (exercise and control), and interaction (group × time) in exercise (*n* = 19) and control (*n* = 14) groups. The dashed line represents the median. Significant difference between pre and post intervention *p < 0.05

SGMS1 increased in the exercise group only (interaction *p* = 0.001; Fig. 6a), with no changes within or between groups for SGMS2 (interaction, *p* = 0.251; Fig. 6b). There was a significant time effect (*p* = 0.008) for ACC, such that ACC increased in the exercise group, without changes in HSL, ATGL, GPAT1, LPCAT3 or HADHSC in either group (Fig. 7). GLUT4 showed a significant time effect (*p* = 0.034) with an increase in response to exercise training (*p* < 0.05; Fig. 8). There were no changes within or between groups for mTOR, PGC-1α, IRS1 and iPLA2γ (Fig. 8).

## Discussion

**Fig. 9:**
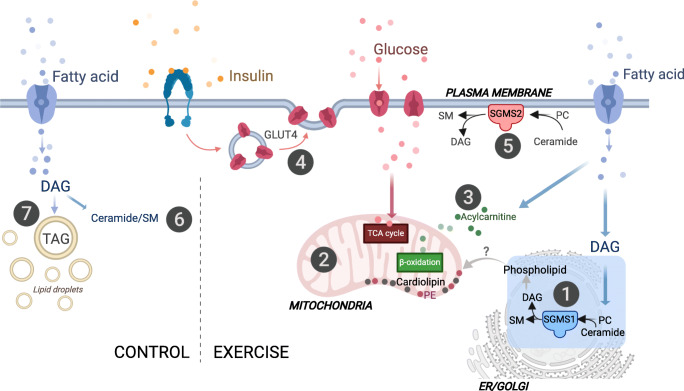
Schematic overview of proposed skeletal muscle pathways affected by the 12 week intervention. (1) Combined aerobic and resistance exercise training resulted in increased SGMS1; sphingolipid metabolism via SGMS1 occurs at the Golgi apparatus through the transfer of a phosphocholine headgroup from PC to ceramide yielding a sphingomyelin (SM) and DAG [17]. (2) Exercise training results in content-driven increases in mitochondrial respiratory capacity that were associated with increases in DAG(32:2), cardiolipins and phospholipids. The increase was more prominent in PE compared with PC, resulting in decreased PC:PE ratios. Both PEs and cardiolipins are highly abundant mitochondrial lipids that contribute to mitochondrial respiratory capacity and content [23, 46, 54]. (3) Exercise training also increased medium-to-long chain acylcarnitines that were associated with increased mitochondrial respiration. Acylcarnitines shuttle fatty acids towards the mitochondria for beta-oxidation. (4) GLUT4 protein expression and whole-body insulin sensitivity increased with exercise training but were not related to change in muscle lipid signatures. (5) The SGMS2 isoform located at the plasma membrane was not affected by the exercise training intervention. The control group increased skeletal muscle total lipid levels of (6) ceramides and SM and (7) TAG, which may have future implications for insulin-desensitising mechanisms [6]. There was no change in mitochondrial respiration or content in the control group. We hypothesise that exercise training increases lipid utilisation in the more bioenergetically active organelles and membranes. Specifically, increased mitochondrial respiration with exercise training may stimulate increases in SGMS1 at the Golgi. SGMS1 produces DAGs that pool in the ER/Golgi network and produce phospholipid intermediates [20]. We propose that these phospholipid intermediates are imported into the mitochondria (via unknown mechanisms) and used as substrates for the synthesis of cardiolipins and PE [21]. This may be a pathway responsible for content-driven improvements in mitochondrial function, while preventing the build-up of DAGs at the plasma membrane where insulin signalling can be perturbed [10, 11, 47]. Created with BioRender.com

The major findings of our study were that exercise training altered specific intramuscular lipid intermediates, which were associated with content-driven increases in mitochondrial respiration, and not whole-body insulin sensitivity. Notably, exercise training did not alter total skeletal muscle lipid content (measured by MRI and MRS), but instead altered specific phospholipids, DAGs, ceramides and acylcarnitines. Additionally, we show increased SGMS1 protein content, not SGMS2, which may indicate increased phospholipid hydrolysis in the Golgi apparatus, rather than at the plasma membrane (Fig. 9). These findings highlight new mechanisms whereby combined aerobic and resistance exercise training may prevent lipotoxicity in skeletal muscle, with putative protection against future type 2 diabetes.

We propose that improved mitochondrial respiratory capacity and content in response to exercise training alters specific phospholipids, DAGs and ceramide, independent of the improvements in insulin sensitivity and GLUT4 protein expression (Fig. 9). This altered lipid signature might, in part, occur via an increase in the sphingomyelin synthase pathway in the Golgi apparatus [9], which is supported by the simultaneous increase in SGMS1 in the exercise group, with no change in SGMS2. SGMS1 is localised in the Golgi apparatus and has previously been related to cell growth, whereas SGMS2 is mainly found at the plasma membrane and has been linked to obesity and insulin resistance [17, 19, 45]. Since the mitochondria rely on lipid influx to maintain membrane integrity and overall cellular function [46], we hypothesise that exercise training increases lipid utilisation in the more bioenergetically active organelles and membranes. Specifically, SGMS1 produces DAGs that pool in the ER/Golgi network and produce phospholipid intermediates [20]. We propose that these phospholipid intermediates are imported into the mitochondria (via unknown mechanisms) and used as substrates for the synthesis of cardiolipins and PE [21]. This may be a pathway responsible for content-driven improvements in mitochondrial function, while preventing the build-up of DAGs at the plasma membrane where insulin signalling may be perturbed [10, 11, 47] (Fig. 9). Notably, the high content of TAGs and phospholipids meant that we were only able to detect a low number of DAGs, with only one, DAG(32:2), changing in response to exercise. Identification of additional DAG species, phospholipid intermediates and organelle-specific proteins involved in the sphingomyelin synthase pathway and the movement of phospholipids to the mitochondria may increase our understanding on potential links between sphingolipid metabolism and mitochondrial respiratory capacity and content (Fig. 9).

Sphingomyelin synthases, via their function in phospholipid hydrolysis, are one of many important regulators of DAGs and ceramides in the tissue [48]. We found that an increase in specific ceramide and DAG species, rather that total content, was associated with increased mitochondrial respiratory capacity, but not with increased whole-body insulin sensitivity or GLUT4 protein content. Ceramides are composed of a sphingosine base, which often contain a d18:1 acyl group, and an amide-linked fatty acyl chain that varies from C14:0–C26:0, with different saturation levels. The variation in fatty acyl composition of ceramides depends on the combination of fatty acyl-CoAs availability and activity of specific ceramide synthases within tissues [10]. Data from human skeletal muscle are sparse, but C24:0 and C24:1-ceramides have been linked to cell proliferation [45], whereas C18-ceramides are linked to increased mitophagy and cell death [45]. Correspondingly, we show that higher concentrations of ceramide(d18:1/18:1) and ceramide(d18:1/22:1) were related to lower mitochondrial respiratory capacity at baseline, and the upregulation of ceramide(d18:1/24:0) with exercise training related to mitochondrial biogenesis. Given that ceramides and PCs are substrates for sphingomyelin synthase, the increase in only ceramide(d18:1/24:0) indicates that other organelle-specific sphingolipid efflux pathways are altered with exercise training [11, 49]. We propose that linking organelle-specific sphingolipid metabolism with mitochondrial biogenesis may assist in finding target mechanisms for the prevention of skeletal muscle lipotoxicity and ensuing type 2 diabetes (Fig. 9).

Sphingolipid metabolism via SGMS1 and SGMS2 occurs through the transfer of a phosphocholine residue from PC to ceramide yielding a sphingomyelin and DAG [17]. Together with PCs, PEs are the most abundant phospholipids in mitochondrial membranes, with the removal of PE causing diminished mitochondrial respiratory capacity in rodents [12, 13]. The PC:PE ratio has shown to be closely linked to skeletal muscle lipid mobilisation and associated with lower insulin sensitivity in overweight men [50, 51]. The current study shows that the increase in PCs and PEs were associated with content-driven changes in mitochondrial function. Moreover, we showed a more prominent increase in PEs that contain 20:4 and 22:5 fatty acyl groups (Fig. 4). This resulted in decreased PC:PE ratios with these fatty acyl groups, which were associated with increased mitochondrial respiratory capacity (Fig. 5). Higher levels of polyunsaturated fatty acyls in phospholipids increase the permeability and fluidity of the cell membrane, which can have direct repercussions on mitochondrial function [22, 23]. Other studies indicate an association between skeletal muscle phospholipids that contain polyunsaturated fatty acyls and insulin sensitivity [52]. Furthermore, 12 weeks of aerobic and resistance training in normal-weight men showed that improved insulin sensitivity was associated with increased total content of skeletal muscle PC (21%) and PE (42%) and reduced total PC:PE ratio (16%) [14]. However, we show no association between altered PC:PE ratios (total content and specific intermediates) and whole-body insulin sensitivity. These discrepancies in outcomes may involve methods used to measure insulin sensitivity and/or differences in exercise training modes.

Interestingly, we showed that exercise training increased the PC:PE(18:1/18:0) ratio, driven by an increase in PC(18:1/18:0). Since de novo synthesis only produces saturated and monounsaturated phospholipids [44], these results may reflect increased demand and de novo synthesis of PCs [53]. The incorporation of polyunsaturated fatty acyls into phospholipids occurs through phospholipid remodelling in the Lands pathway, via their deacylated lyso-form (i.e. LPE and LPC) by PLA2, followed by reacylation and incorporation of a polyunsaturated fatty acid via LPCAT [44]. In addition to the observed increase in PE(18:0/22:5) following exercise training, we show an increase in its corresponding lyso-form, LPE(22:5). This implies that exercise training impacts phospholipid remodelling, and specifically remodelling of PEs, which occurs via various PLA2s and LPCATs [44]. Although we show no changes in iPLA2γ and LPCAT3 protein content, there are a vast number of unexplored organelle-specific phospholipase and acyltransferases that could be affected and stimulate changes in phospholipid acyl composition. Further research is important to understand the role of specific phospholipases and acyltransferases in regulating specific skeletal muscle phospholipid intermediates and potentially also insulin sensitivity.

We showed that an increase in skeletal muscle cardiolipins were associated with an increase in content-driven changes in mitochondrial function. Cardiolipins play a central role in mitochondrial respiration and energy production and are uniquely located in the mitochondrial membranes [23, 54]. Interestingly, alterations in the phospholipid composition can also affect membrane integrity, permeability and transport, and the cardiolipin acyl groups may be important when understanding this link [23, 54]. The cardiolipins that increased in the current study occurred predominately in those with a higher degree of unsaturation (total double bonds >7) and correlated with the increase in mitochondrial respiratory capacity. Similarly, cell culture studies have shown correlations between increased cardiolipins with a higher degree of unsaturation, and improved mitochondrial activity and reduced inflammation [22]. Regardless, the increase in cardiolipins with a high degree of unsaturation observed in response to exercise training may indicate an improved membrane permeability and transportation, which is essential for stimulating mitochondrial biogenesis.

The long-term exposure to a build-up of fatty acids and BCAA intermediates is considered ‘lipotoxic’ and has been shown to contribute to skeletal muscle insulin resistance and the pathogenesis of type 2 diabetes [55]. At baseline, our study showed higher hydroxylated acylcarnitines related to higher mitochondrial respiratory capacity and not whole-body insulin sensitivity or GLUT4. Hydroxylated acylcarnitines have been previously observed during dysfunctional mitochondrial fatty acid oxidation and may simply reflect an oversupply of fatty acids to the peripheral tissue prior to the intervention [56]. Alternatively, skeletal muscle acylcarnitines increase in response to exercise training and relate to mitochondrial remodelling and cardiometabolic fitness [57]. Similarly, we found that exercise training stimulated an increase in the fatty-acid-related acylcarnitines (C12- to C20-carnitines), and a concomitant decrease in acylcarnitines related to BCAA catabolism (C3-, C5-carnitines). These changes were associated with improved mitochondrial respiration (Fig. 5) rather than changes in insulin sensitivity. Accordingly, we suggest that the increased capacity for mitochondrial fatty acid oxidation and content may prevent the accumulation of BCAA catabolic intermediates. Altered BCAA metabolism in the liver and adipose tissue contributes to an obesity-related elevation in circulating BCAA that ultimately interferes with lipid oxidation in the skeletal muscle [58, 59]. Accordingly, tissue-specific measures of insulin sensitivity (hepatic and/or peripheral) in relation to skeletal muscle BCAAs may be of interest to explore.

The high risk for weight gain in young South African women [27] was further supported by the results in the control group, who gained weight (±1 kg) over the 12 week period. The control group also showed an increase in skeletal muscle lipid intermediates, such as TAGs, ceramides and sphingomyelins (Fig. 9). Although the exact mechanism is unclear, we hypothesise that the increase in ACC protein expression, with no increases in mitochondrial respiration or content, may favour the formation of fatty acids through malonyl-CoA and the overall storage of lipids. A build-up of NEFA can lead to the inhibition of GLUT4 and contribute to the development of skeletal muscle insulin resistance [60]. The current study shows no association at baseline or in response to the intervention between the lipid signature and GLUT4; we suggest that an inverse relationship may be evident in a cohort with insulin resistance or diabetes.

In conclusion, exercise training in women with obesity altered intramuscular phospholipid, cardiolipin, acylcarnitine, DAG and ceramide subtypes that were associated with content-driven changes in mitochondrial respiration, but not whole-body insulin sensitivity. We propose that exercise training increases lipid utilisation in the more bioenergetically active organelles and membranes, which may prevent future skeletal muscle lipotoxicity and this may relate to hepatic and/or peripheral estimates of insulin sensitivity. Further interventions designed to specifically manipulate muscle lipids and/or mitochondrial function (i.e. diet and/or different exercise training modes) are required to understand whether changes in muscle lipids are fundamental to support the growth and morphology of a mitochondrial network (interdependent geometrical feature and their dynamics), rather than mitochondrial function per se.

## Supplementary Information

ESM (PDF 531 kb)

## Data Availability

The datasets generated during and/or analysed during the current study are available from the corresponding author on reasonable request.

## Abbreviations

ACC: Acetyl-CoA carboxylase
ATGL: Adipose triglyceride lipase
BCAA: Branched-chain amino acid
CV-ANOVA: ANOVA of the cross-validated OPLS scores
DAG: Diacylglycerol
DXA: Dual-energy x-ray absorptiometry
EMCL: Extra-myocellular lipid content
ER: Endoplasmic reticulum
ETF: Electron transferring flavoprotein
ETF^p^: Lipid OXPHOS capacity
ETS: Electron transport system
GPAT1: Glycerol-3-phosphate acyltransferase 1
HADHSC: Hydroxyacyl-CoA dehydrogenase
HR_peak_: Peak heart rate
HSL: Hormone sensitive lipase
IMCL: Intra-myocellular lipid content
iPLA2γ: Phospholipase A2γ
Leak^ETF^: Lipid-induced leak respiration
Leak^Oly^: Oligomycin-induced leak respiration
LPC: Lysophosphatidylethanolamine
LPCAT: Lysophosphatidylethanolamine acyltransferase
LPE: Lysophosphatidylethanolamine
MRS: Magnetic resonance spectroscopy
mTOR: Mammalian target of rapamycin
OPLS: Orthogonal partial least squares
OPLS-EP: Orthogonal partial least squares-effect projections
OXPHOS: Oxidative phosphorylation
PC: Phosphatidylcholine
PE: Phosphatidylethanolamine
PGC-1α: PPARG coactivator 1α
S_I_: Insulin sensitivity index
SGMS1: Sphingomyelin synthase 1
SGMS2: Sphingomyelin synthase 2
TAG: Triacylglycerol
TCA: Tricarboxylic acid cycle
w.w.: Wet weight
