# Supplementary material for: Exercise training improves mitochondrial respiration and is associated with an altered intramuscular phospholipid signature in women with obesity
Source: Diabetologia. 2021 Mar 26;64(7):1642–59. doi: 10.1007/s00125-021-05430-6 (PMC8187207; doi:10.1007/s00125-021-05430-6)
Supplement: Supplementary file 1 — (PDF 531 kb) [file 125_2021_5430_MOESM1_ESM.pdf]

ESM Methods

Muscle preparation and western blotting

Portions (20 mg) of the muscle biopsies were homogenized with a TissueLyser II (QIAGEN, Hilden, Germany), in 0.6 mL of ice-cold lysis buffer (137 mmol/l NaCl, 1 mmol/l $MgCl_2$, 2.7 mmol/L KCl, 1 mmol/L EDTA, 20 mmol/l Tris, pH 7.8, 5 mmol/l Na pyrophosphate, 10 mmol/l NaF, 1% Triton X-100, 10% [vol/vol] glycerol, 0.2 mmol/l phenylmethylsulfonyl fluoride [PMSF], 0.5 mmol/l Na_3VO_4 and 1× protease inhibitor cocktail Set 1 [Calbiochem, EMD Biosciences, San Diego, CA, USA]). Insoluble material was removed by centrifugation at 12 000 g for 10 min at 4°C, and supernatant protein concentration was determined using a commercially available assay (Pierce BCA protein assay kit; Thermo Scientific, Rockford, IL, USA). Equal amounts of protein were diluted in Laemmli buffer, separated by SDS-PAGE electrophoresis (Criterion XT Precast gel; Bio-Rad, Hercules, CA, USA) and were transferred to PVDF membranes. Equal loading was confirmed by Ponceau S staining. The membranes were blocked with 5% non-fat dry milk in TBST (20 mmol/l Tris, 137 mmol/l NaCl, 0.02% Tween 20, pH 7.6) for 1 h at room temperature and incubated overnight at 4°C with appropriate primary antibodies diluted 1:1000 in TBS with 0.1% BSA and 15 mmol/l NaN_3. Membranes were washed in TBST and incubated with the respective secondary antibodies diluted in 5% non-fat dry milk in TBST, as recommended by the supplier (Amersham, Arlington, IL, USA). Proteins were visualized by enhanced chemiluminescence (Amersham) and quantified by densitometry using Quantity One software (Bio-Rad).

Mitochondrial respiratory capacity

Measures of mitochondrial respiration were performed in respiration medium (MiR05) at 37°C using high-resolution respirometry (Oxygraph-2k; Oroboros, Innsbruck, Austria). All measures were completed in duplicate and carried out in a hyperoxygenated (250-450 nmol/ml) environment. Skeletal muscle samples were prepared and analyzed according to previously described methods [1]. Briefly, immediately after tissue collection, samples were stored in ice-cold BIOPS [1] for a maximum of 4 h before analyses. Skeletal muscle (2-3 mg w.w) were permeabilized in saponin (50 μg/mL BIOPS) for 30 min and washed in MiR05 for 2 x 10 min. The multiple SUIT protocol included [2, 3]: (1) Lipid-induced respiration through the electron transferring flavoprotein (ETF) in the absence of adenylates ($Leak^{ETF}$) with the addition of malate (2 mM) and octanoyl-carnitine (0.2 mM); (2) Lipid OXPHOS

capacity (ETF^p) with the addition of ADP (5 mM); (3) State 3 respiration capacity (Complex I) specific to ETF and complex I (pyruvate 5 mM; glutamate 10 mM); (4) Maximal state 3 respiration (Complex I+II), oxidative phosphorylation capacity (Succinate, 10 mM); (5) State 4o respiration, oligomycin-induced leak respiration ($Leak^{oly}$) through inhibition of ATP synthase (Oligomycin 2.5 μM); (6) Electron transports system (ETS) capacity with the titration of carbonyl cyanide *m*-chlorophenyl hydrazone (0.5 μM titration steps); (7) Inhibition of complex I with the addition of rotenone (0.5 μM); (8) The inhibition of complex III with the addition of antimycin A (2.5 μM). Complex III inhibition was used for the determination and correction of residual oxygen consumption (non-mitochondrial oxygen consumption in the chamber). All respiration data are reported relative to mass (pmol s^{-1} [mg w.w.]$^{-1}$) and mitochondrial content (pmol s^{-1} [mg w.w.]$^{-1}$/CS protein content [AU]). Citrate synthase (CS) protein content was used as a marker of mitochondrial content and divided into mass specific respiration to calculate content specific mitochondrial respiration, which is termed intrinsic mitochondrial function.

Metabolomic and lipidomic analyses

Skeletal muscle samples were extracted with methanol/water (90/10, vol/vol) for metabolomic analyses and chloroform/methanol (2:1, vol/vol) for lipidomic analyses, both extraction mixtures are spiked with isotope labelled internal standards. Samples were prepared and analysed in designed batches to reduce the methodological biases that may interfere with the interpretation of the results [4], i.e., samples from the same individual were prepared and analyzed in close connection, but with a randomized internal order. In addition, analytical batches were balanced in terms of treatment group and quality control samples (i.e., pooled from all samples, blanks and injection standards) were added to monitor instrument stability.

Prior to GC-TOF/MS metabolomics analysis, a further two-step derivatization procedure was carried out to increase metabolites volatility (silylated) and reduce number of tautomeric forms of each carbohydrate (methoxymated). The derivatized samples (1 μl) were analyzed on an Agilent 6890 gas chromatograph equipped with a 10 m × 0.18 mm i.d. fused silica capillary column with a chemically bonded 0.18-μm DB 5-MS stationary phase (J&W Scientific, Folsom, CA). The injector temperature was 270°C. The column temperature was held at 70°C for 2 min, increased by 40°C min^{-1} to 320°C, and held there for 1 min. The column effluent was introduced into the ion source of a Pegasus III time-

of-flight mass spectrometer, GC-TOF/MS (Leco, St. Joseph, MI, USA). The transfer line and the ion source temperatures were 250 and 200°C, respectively. Ions were generated by a 70-eV electron beam at an ionization current of 2.0 mA. The spectra were recorded in the mass range 50–800 m/z at a rate of 30 spectra s^{-1}.

Prior to LC-TOF/MS analyses, the samples were re-suspended in 10 + 10 µl of methanol and water for metabolomic analysis and re-suspended in chloroform/methanol (2:1, vol/vol) for lipidomic analysis. Aliquots of muscle extracts (2 µl) were injected onto a Waters Acquity UPLC HSS T3 C_{18} column (2.1 × 50 mm, 1.8 µM, Waters, Milford, MA, USA) in combination with a 2.1 mm x 5 mm, 1.8 µm VanGuard precolumn (Waters Corporation, Milford, MA, USA) held at 40°C. The chromatographic separations of metabolites were carried out using a gradient solvent system consisted of water with 0.1% formic acid (A) and acetonitrile/isopropanol (75/25, vol/vol) with 0.1% formic acid (B) at a flow rate of 0.5 ml min^{-1}. The gradient started at 0.1% B increasing linearly to 10% B in 2 min and increased to 99% B over 5 min which held for 2 min; the proportion of B was decreased to 0.1% for 0.3 min and was further decreased to 0% with the flowrate increased to 0.8 ml min^{-1} for 0.5 min which held for 0.9 min; the initial conditions were restored in 0.1 min before the next injection.

The chromatographic separations of lipids (aliquots of muscle lipid extracts 0.5 µl) were carried out using a gradient solvent system consisted of mobile phase A (60:40 acetonitrile:water + 10 mM ammonium formate + 0.1% formic Acid) and mobile phase B (89.1:10.5:0.4 isopropanol:acetonitrile:water + 10 mM ammonium formate + 0.1% formic acid) at a flow rate of 0.5 ml min^{-1}. The gradient started at 15% B increasing linearly to 30% B in 1.2 min and increased to 55% B over 0.3 min which held for 3.5 min; the proportion of B was increased to 72% in 2 min and increased to 85% in 2.5 min, and further increased to 100% in 0.5 min. The wash program started after the sample analysis; the initial conditions were restored in 0.3 min before the next injection.

The detection of separated metabolites and lipids was performed using the Agilent 6550 Q-TOF mass spectrometer equipped with a jet stream electrospray ionization (ESI) source, operating in both positive and negative ion modes. A reference interface was connected for accurate mass measurements and reproducibility. Full scan MS spectra were collected in a centroid mode over the

mass range 70-1700 m/z with an acquisition rate of 4 spectra s^{-1}. The capillary voltage was set at +4 kV and –4 kV with nozzle voltages of +300 V and –300V for positive and negative ion modes, respectively. Other MS parameters were applied as follows: gas temperature was set at 150°C, drying gas flow was 16 l min^{-1}, and the pressure of nebulizer gas was 35 psig. The sheath gas flow was kept at 11 L $min^{-1}$with a temperature of 350°C. The voltages of the fragmentor, skimmer, and octopole RF peak voltage were 380 V, 45 V and 750 V, respectively. The LC-TOF/MS data acquisition was performed in global profiling mode including the initial MS/MS scanning for the further targeted and untargeted data analysis. Additional targeted and untargeted MS/MS analyses of quality control samples were performed for targeting metabolites included in the in-house database and for elucidating the structures of untargeted metabolites, respectively. Data were acquired with MassHunter Acquisition Software B.05.01.

The processing of GC-TOF/MS data and extraction of putative metabolites are described by Chorell et al [5]. Briefly, an in-house MATLAB script was used for the extraction of putative metabolites by matching the mass spectra and retention indices to in-house mass spectral library at the Swedish Metabolomics Centre and the publicly available Max Planck Institute library in Golm. The processing of LC-TOF/MS data and extraction of putative metabolites and lipids were performed by MassHunter Profinder version B.08.00 in combination with Qualitative Analysis software version B.07.00, PCDL manager version B.07.00 and Mass Profiler Professional™ 13.0 (all from Agilent Technologies Inc., Santa Clara, CA, USA). Annotation of putative metabolites were done by matching the retention time (MS and MS-MS spectra) against the in-house metabolite and lipid library. The match tolerance of masses deviation was set to ± 20 ppm and retention time deviation was adjusted to ± 0.15 min, respectively. Features were firstly filtered by manual inspection of the data chromatographic and mass spectral profiles and removal of non-Gaussian peaks as well as peaks with deviating % RSD-values > 10 ppm (Mass) and % RSD > 20 (target score of molecular feature extraction). Next, to remove noisy features and background, we calculated an OPLS model for the extracted features using the concentration of the dilution series of QC samples as response. Features with VIP < 1 and with a negative correlation to the dilution series of QC samples were removed. By using metabolic profiling mode, numerous of unknown putative metabolites and lipids, not available in current databases, could be quantified and included in sample comparison modelling.

Based on the above data analysis strategy and criteria, only a few DAG species were detected due to the large amount of triacylglyceride content which forced us to reduce injection of muscle lipid extracts from 1 to 0.5 µl to prevent peak saturation mainly caused by phospholipids and triacylglycerols. Due to the low abundance of diacylglycerols (DAG), we were not able to identify the exact individual fatty acyl moieties of the DAGs by LC-MS/MS analyses.

ESM Results

ESM Table 1. List of identified metabolites in the muscle samples of control and exercise groups

ID	Metabolite class	Metabolite name	Abbr.	Lipids Identities	Platform	HMDB ID	CAS No.	Altered with exercise (OPLS, CV- ANOVA P<0.05)
G140	Fatty acid	Nonanoic acid	FA(C9:0)		GC-MS Metabolomics			↓
G143	Fatty acid	Pentadecanoic acid	FA(C15:0)		GC-MS Metabolomics	HMDB00826		↓
G139	Fatty acid	Hexadecanoic acid	FA(C16:0)		GC-MS Metabolomics	HMDB00220	57-10-3	↓
L243	Fatty acid	Hydroxycaprylic acid (8:0-OH)	FA(C8:0-OH)		LC-MS Metabolomics	HMDB02264		↓
L249	Fatty acid	Sebacic acid (C10:0-DC)	FA(C10:0-DC)		LC-MS Metabolomics	HMDB00792		↓
G138	Fatty acid	3-Hydroxylauric acid	FA(C12:0-(3-OH))		GC-MS Metabolomics			↑
L252	Fatty acid	Myristic acid (14:0)	FA(C14:0)		LC-MS Metabolomics	HMDB00806	544-63-8	↓
L255	Fatty acid	cis-9-palmitoleic acid (16:1)	FA(C16:1)		LC-MS Metabolomics	HMDB03229	373-49-9	↑
L256	Fatty acid	Palmitic acid (16:0)	FA(C16:0)		LC-MS Metabolomics	HMDB00220	57-10-3	↓
L258	Fatty acid	β-Hydroxypalmitic acid (16:0-OH)	FA(C16:0(3-OH))		LC-MS Metabolomics	HMDB61658	928-17-6	↓
L259	Fatty acid	Hydroxyhexadecanoic acid (16:0-OH)	FA(C16:0-OH)		LC-MS Metabolomics	HMDB62548	764-67-0	↓
L260	Fatty acid	Linoleic acid (18:2)	FA(C18:2)		LC-MS Metabolomics	HMDB00673	60-33-3	↓
G141	Fatty acid	9, 12-Octadecadienoic acid	FA(C18:2)		GC-MS Metabolomics	HMDB00673	60-33-3	↑
L261	Fatty acid	Oleic acid (18:1)	FA(C18:1)		LC-MS Metabolomics	HMDB00207	112-79-8	↓
G142	Fatty acid	Octadecanoic acid	FA(C18:0)		GC-MS Metabolomics	HMDB00827	57-11-4	↓
L262	Fatty acid	Stearic acid (18:0)	FA(C18:0)		LC-MS Metabolomics	HMDB00827	57-11-4	↓
L271	Ceramide	Cer(d18:1/18:0)	Cer(d18:1/18:0)		LC-MS Lipidomics	HMDB0004950		↑
L272	Ceramide	Cer(d18:1/24:0)	Cer(d18:1/24:0)		LC-MS Lipidomics	HMDB0004956		↑
L273	Ceramide	Cer(d18:1/18:1)	Cer(d18:1/18:1)		LC-MS Lipidomics	HMDB0004948		↑
L274	Ceramide	Cer(d18:1/22:1)	Cer(d18:1/22:1)		LC-MS Lipidomics			↑
L275	Ceramide	Cer(d18:1/24:1)	Cer(d18:1/24:1)		LC-MS Lipidomics	HMDB0004953		↑
L276	Ceramide	GalCer(d18:1/18:0)	GalCer(d18:1/18:0)		LC-MS Lipidomics			↓
L277	Ceramide	GalCer(d18:1/20:3)	GalCer(d18:1/20:3)		LC-MS Lipidomics			↑
L278	Diacylglycerol	DAG(32:2)	DG(32:2)		LC-MS Lipidomics			↑
L279	Diacylglycerol	DAG(32:3)	DG(32:3)		LC-MS Lipidomics			↑
L280	Diacylglycerol	DAG(34:3)	DG(34:3)		LC-MS Lipidomics			↓
L281	Diacylglycerol	DAG(38:4)	DG(38:4)		LC-MS Lipidomics			↑
L282	Diacylglycerol	DAG(33:5)	DG(33:5)		LC-MS Lipidomics			↑
L1	Lysophosphatidylcholine	LPC(18:1)	LPC(18:1)		LC-MS Lipidomics	HMDB10385		↓
L2	Lysophosphatidylcholine	LPC(18:2)	LPC(18:2)		LC-MS Lipidomics	HMDB10386		↑
L211	Lysophosphatidylcholine	LPC(14:0)	LPC(14:0)		LC-MS Metabolomics	HMDB10379		↓
L212	Lysophosphatidylcholine	LPC(P-16:0) or LPC(O-16:1)	LPC(P-16:0) or LPC(O-16:1)		LC-MS Metabolomics			↓
L213	Lysophosphatidylcholine	LPC(O-16:0)	LPC(O-16:0)		LC-MS Metabolomics		52691-62-0	↓
L214	Lysophosphatidylcholine	LPC(16:1)	LPC(16:1)		LC-MS Metabolomics	HMDB10383		↓
L215	Lysophosphatidylcholine	LPC(16:0)	LPC(16:0)		LC-MS Metabolomics	HMDB10382		↓
L216	Lysophosphatidylcholine	LPC(17:0)	LPC(17:0)		LC-MS Metabolomics	HMDB12108		↓
L217	Lysophosphatidylcholine	LPC(18:3)	LPC(18:3)		LC-MS Metabolomics	HMDB10388		↓
L218	Lysophosphatidylcholine	LPC(18:2)	LPC(18:2)		LC-MS Metabolomics	HMDB10386		↓
L219	Lysophosphatidylcholine	LPC(18:1)	LPC(18:1)		LC-MS Metabolomics	HMDB10385		↓
L220	Lysophosphatidylcholine	LPC(18:0)	LPC(18:0)		LC-MS Metabolomics	HMDB10384		↓
L221	Lysophosphatidylcholine	LPC(20:4)	LPC(20:4)		LC-MS Metabolomics	HMDB10395		↓
L222	Lysophosphatidylcholine	LPC(20:3)	LPC(20:3)		LC-MS Metabolomics	HMDB10393		↓
L265	Lysophosphatidylethanolamine	LPE(16:0)	LPE(16:0)		LC-MS Metabolomics	HMDB11503		↓
L266	Lysophosphatidylethanolamine	LPE(18:2)	LPE(18:2)		LC-MS Metabolomics	HMDB11507		↓
L267	Lysophosphatidylethanolamine	LPE(18:1)	LPE(18:1)		LC-MS Metabolomics	HMDB11506		↓
L268	Lysophosphatidylethanolamine	LPE(18:0)	LPE(18:0)		LC-MS Metabolomics	HMDB11130		↑
L269	Lysophosphatidylethanolamine	LPE(22:5)	LPE(22:5)		LC-MS Metabolomics	HMDB0011519		↑
L3	Phosphatidylcholine	PC(30:0)	PC(30:0)		LC-MS Lipidomics			↑
L4	Phosphatidylcholine	PC(31:0)	PC(31:0)		LC-MS Lipidomics			↑
L5	Phosphatidylcholine	PC(32:0)	PC(32:0)		LC-MS Lipidomics			↑
L6	Phosphatidylcholine	PC(32:1)	PC(32:1)	6:0/16:1) or PC(14:0	LC-MS Lipidomics			↑
L7	Phosphatidylcholine	PC(32:2)	PC(32:2)	PC(14:0/18:2)	LC-MS Lipidomics			↑
L8	Phosphatidylcholine	PC(33:1)	PC(33:1)		LC-MS Lipidomics			↑
L9	Phosphatidylcholine	PC(33:2)	PC(33:2)		LC-MS Lipidomics			↑
L10	Phosphatidylcholine	PC(34:0)	PC(34:0)		LC-MS Lipidomics			↑
L11	Phosphatidylcholine	PC(34:1)	PC(34:1)	PC(16:0/18:1)	LC-MS Lipidomics	HMDB0007972		↑
L12	Phosphatidylcholine	PC(34:2)	PC(34:2)	PC(16:0/18:2)	LC-MS Lipidomics			↑
L13	Phosphatidylcholine	PC(34:3)	PC(34:3)	PC(16:1/18:2)	LC-MS Lipidomics	HMDB0008006		↑
L14	Phosphatidylcholine	PC(35:2)	PC(35:2)		LC-MS Lipidomics			↑
L15	Phosphatidylcholine	PC(35:3)	PC(35:3)		LC-MS Lipidomics			↑
L16	Phosphatidylcholine	PC(36:1)	PC(36:1)	PC(18:1/18:0)	LC-MS Lipidomics	HMDB0008069		↑
L17	Phosphatidylcholine	PC(36:2)	PC(36:2)	PC(18:2/18:0)	LC-MS Lipidomics	HMDB0008135		↑
L18	Phosphatidylcholine	PC(36:3)	PC(36:3)	PC(18:1/18:2)	LC-MS Lipidomics	HMDB0008105		↑
L19	Phosphatidylcholine	PC(36:4)	PC(36:4)	PC(18:0/20:4)	LC-MS Lipidomics	HMDB0008048		↑
L20	Phosphatidylcholine	PC(36:5)	PC(36:5)	PC(16:0/20:5)	LC-MS Lipidomics	HMDB0007984		↑
L21	Phosphatidylcholine	PC(38:3)	PC(38:3)		LC-MS Lipidomics			↑

L22	Phosphatidylcholine	PC(38:4)	PC(38:4)	LC-MS Lipidomics		↑
L23	Phosphatidylcholine	PC(38:5)	PC(38:5)	LC-MS Lipidomics		↑
L24	Phosphatidylcholine	PC(38:6)	PC(38:6)	PC(16:0/22:6) LC-MS Lipidomics		↑
L25	Phosphatidylcholine	PC(40:5)	PC(40:5)	LC-MS Lipidomics		↓
L26	Phosphatidylcholine	PC(40:7)	PC(40:7)	LC-MS Lipidomics		↑
L27	Phosphatidylcholine	PC(O-34:2)	PC(O-34:2)	PC(O-18:1/16:1) LC-MS Lipidomics		↑
L28	Phosphatidylcholine	PC(O-34:3)	PC(O-34:3)	PC(O-16:1/18:2) LC-MS Lipidomics	HMDB0013413	↑
L29	Phosphatidylcholine	PC(O-36:4)	PC(O-36:4)	PC(O-18:2/18:2) LC-MS Lipidomics	HMDB0013435	↑
L30	Phosphatidylcholine	PC(O-36:5)	PC(O-36:5)	PC(O-16:1/20:4) LC-MS Lipidomics		↑
L31	Phosphatidylcholine	PC(O-36:6)	PC(O-36:6)	LC-MS Lipidomics		↓
L32	Phosphatidylcholine	PC(O-38:5)	PC(O-38:5)	LC-MS Lipidomics		↑
L33	Phosphatidylcholine	PC(O-38:6)	PC(O-38:6)	PC(O-16:1/22:5), P LC-MS Lipidomics		↑
L34	Phosphatidylcholine	PC(O-38:7)	PC(O-38:7)	LC-MS Lipidomics		↑
L130	Phosphatidylethanolamine	PE(34:1)	PE(34:1)	LC-MS Lipidomics		↓
L131	Phosphatidylethanolamine	PE(36:1)	PE(36:1)	PE(18:1/18:0) LC-MS Lipidomics	HMDB0009057	↑
L132	Phosphatidylethanolamine	PE(34:2)	PE(34:2)	LC-MS Lipidomics		↑
L133	Phosphatidylethanolamine	PE(36:2)	PE(36:2)	PE(18:0/18:2) LC-MS Lipidomics	HMDB0008994	↑
L134	Phosphatidylethanolamine	PE(36:4)	PE(36:4)	PE(16:0/20:4) LC-MS Lipidomics	HMDB0008937	↑
L135	Phosphatidylethanolamine	PE(38:4)	PE(38:4)	PE(18:0/20:4) LC-MS Lipidomics	HMDB0009003	↑
L136	Phosphatidylethanolamine	PE(38:5)	PE(38:5)	PE(18:1/20:4) LC-MS Lipidomics	HMDB0009069	↑
L137	Phosphatidylethanolamine	PE(40:5)	PE(40:5)	PE(18:0/22:5) LC-MS Lipidomics		↑
L138	Phosphatidylethanolamine	PE(O-38:5)	PE(O-38:5)	LC-MS Lipidomics		↑
L139	Phosphatidylethanolamine	PE(40:6)	PE(40:6)	PE(18:2/20:4) LC-MS Lipidomics	HMDB0009102	↑
L141	Phosphatidylinositol	PI(34:1)	PI(34:1)	LC-MS Lipidomics		↓
L142	Phosphatidylinositol	PI(36:1)	PI(36:1)	LC-MS Lipidomics		↓
L143	Phosphatidylinositol	PI(36:2)	PI(36:2)	PI(18:0/18:2) LC-MS Lipidomics	HMDB0009809	↑
L144	Phosphatidylinositol	PI(38:2)	PI(38:2)	LC-MS Lipidomics		↑
L145	Phosphatidylinositol	PI(36:3)	PI(36:3)	PI(18:1/18:2) LC-MS Lipidomics	HMDB0009838	↓
L146	Phosphatidylinositol	PI(38:3)	PI(38:3)	PI(18:0/20:3) LC-MS Lipidomics	HMDB0009814	↑
L147	Phosphatidylinositol	PI(36:4)	PI(36:4)	PI(16:0/20:4) LC-MS Lipidomics	HMDB0009789	↑

L148	Phosphatidylinositol	PI(38:4)	PI(38:4)	PI(18:0/20:4)	LC-MS Lipidomics	HMDB0009815		↑
L149	Phosphatidylinositol	PI(40:4)	PI(40:4)		LC-MS Lipidomics			↑
L150	Phosphatidylinositol	PI(38:5)	PI(38:5)	PI(18:1/20:4)	LC-MS Lipidomics	HMDB0009844		↑
L151	Phosphatidylinositol	PI(40:5)	PI(40:5)	PI(18:0/22:5)	LC-MS Lipidomics			↑
L35	Sphingomyelin	SM(d18:0/16:0)	SM(d18:0/16:0)		LC-MS Lipidomics	HMDB0062665		↑
L36	Sphingomyelin	SM(d18:0/16:1)	SM(d18:0/16:1)		LC-MS Lipidomics			↑
L37	Sphingomyelin	SM(d18:0/18:1)	SM(d18:0/18:1)		LC-MS Lipidomics			↑
L38	Sphingomyelin	SM(d18:1/14:0)	SM(d18:1/14:0)		LC-MS Lipidomics	HMDB0012097		↑
L39	Sphingomyelin	SM(d18:1/16:0)	SM(d18:1/16:0)		LC-MS Lipidomics	HMDB0061712		↑
L40	Sphingomyelin	SM(d18:1/18:0)	SM(d18:1/18:0)		LC-MS Lipidomics	HMDB0062559		↑
L41	Sphingomyelin	SM(d18:1/18:1)	SM(d18:1/18:1)		LC-MS Lipidomics			↑
L42	Sphingomyelin	SM(d18:1/22:0)	SM(d18:1/22:0)		LC-MS Lipidomics	HMDB0012103		↑
L43	Sphingomyelin	SM(d18:1/24:1)	SM(d18:1/24:1)		LC-MS Lipidomics			↑
L44	Sphingomyelin	SM(d18:1/24:2)	SM(d18:1/24:2)		LC-MS Lipidomics			↑
L46	Triacylglycerol	TAG(40:1)	TAG(40:1)	16:0/18:1/6:0	LC-MS Lipidomics			↑
L47	Triacylglycerol	TAG(42:0)	TAG(42:0)		LC-MS Lipidomics			↑
L48	Triacylglycerol	TAG(42:1)	TAG(42:1)	18:1/12:0 or 16:0/18	LC-MS Lipidomics			↑
L49	Triacylglycerol	TAG(44:1)	TAG(44:1)	8:1/10:0 or 16:0/12:	LC-MS Lipidomics			↑
L50	Triacylglycerol	TAG(44:2)	TAG(44:2)	6:0/10:0 or 18:2/14:	LC-MS Lipidomics			↑
L51	Triacylglycerol	TAG(45:1)	TAG(45:1)		LC-MS Lipidomics			↑
L52	Triacylglycerol	TAG(46:0)	TAG(46:0)		LC-MS Lipidomics			↑
L53	Triacylglycerol	TAG(46:3)	TAG(46:3)	or 18:2/10:0/18:1 or	LC-MS Lipidomics			↑
L54	Triacylglycerol	TAG(46:4)	TAG(46:4)		LC-MS Lipidomics			↑
L55	Triacylglycerol	TAG(47:0)	TAG(47:0)		LC-MS Lipidomics			↑
L56	Triacylglycerol	TAG(47:1)	TAG(47:1)		LC-MS Lipidomics			↑
L57	Triacylglycerol	TAG(47:2)	TAG(47:2)		LC-MS Lipidomics			↑
L58	Triacylglycerol	TAG(47:3)	TAG(47:3)		LC-MS Lipidomics			↑
L59	Triacylglycerol	TAG(48:0)	TAG(48:0)		LC-MS Lipidomics			↑
L60	Triacylglycerol	TAG(48:1)	TAG(48:1)		LC-MS Lipidomics			↑
L61	Triacylglycerol	TAG(48:4)	TAG(48:4)	8:2/12:0 or 18:2/16:	LC-MS Lipidomics			↑
L62	Triacylglycerol	TAG(49:0)	TAG(49:0)		LC-MS Lipidomics			↑
L63	Triacylglycerol	TAG(49:1)	TAG(49:1)		LC-MS Lipidomics			↑
L64	Triacylglycerol	TAG(49:2)	TAG(49:2)		LC-MS Lipidomics			↑
L65	Triacylglycerol	TAG(49:3)	TAG(49:3)		LC-MS Lipidomics			↑
L66	Triacylglycerol	TAG(50:1)	TAG(50:1)	16:0/16:0/18:1	LC-MS Lipidomics	HMDB05360		↑
L67	Triacylglycerol	TAG(50:2)	TAG(50:2)	or 18:1/14:0/18:1 or	LC-MS Lipidomics			↑
L68	Triacylglycerol	TAG(50:4)	TAG(50:4)	4:0/18:2 or 18:2/16:	LC-MS Lipidomics			↑
L69	Triacylglycerol	TAG(50:5)	TAG(50:5)	6:1/16:1 or 18:2/18:	LC-MS Lipidomics			↑
L70	Triacylglycerol	TAG(51:0)	TAG(51:0)		LC-MS Lipidomics			↑
L71	Triacylglycerol	TAG(51:1)	TAG(51:1)	8:1/17:0 or 18:1/15:	LC-MS Lipidomics			↑
L72	Triacylglycerol	TAG(51:2)	TAG(51:2)	or 18:2/17:0/16:0 or	LC-MS Lipidomics			↑
L73	Triacylglycerol	TAG(51:3)	TAG(51:3)	6:1/17:1 or 18:2/15:	LC-MS Lipidomics			↓
L74	Triacylglycerol	TAG(51:4)	TAG(51:4)		LC-MS Lipidomics			↓
L75	Triacylglycerol	TAG(52:2)	TAG(52:2)	16:0/18:1/18:1	LC-MS Lipidomics	HMDB05382		↑
L76	Triacylglycerol	TAG(52:3)	TAG(52:3)	6:0/18:2 or 18:1/16:	LC-MS Lipidomics			↑
L77	Triacylglycerol	TAG(52:4)	TAG(52:4)	6:0/18:2 or 18:2/18:	LC-MS Lipidomics			↑
L78	Triacylglycerol	TAG(52:5)	TAG(52:5)	6:0/18:3 or 16:1/18:	LC-MS Lipidomics			↑
L79	Triacylglycerol	TAG(52:6)	TAG(52:6)	18:3/18:2/16:1	LC-MS Lipidomics			↑
L80	Triacylglycerol	TAG(52:7)	TAG(52:7)		LC-MS Lipidomics			↑
L81	Triacylglycerol	TAG(53:1)	TAG(53:1)		LC-MS Lipidomics			↑
L82	Triacylglycerol	TAG(53:2)	TAG(53:2)	7:0/18:1 or 18:1/19:	LC-MS Lipidomics			↑
L83	Triacylglycerol	TAG(53:3)	TAG(53:3)	7:0/18:2 or 18:1/17:	LC-MS Lipidomics			↓
L84	Triacylglycerol	TAG(53:4)	TAG(53:4)		LC-MS Lipidomics			↓
L85	Triacylglycerol	TAG(54:2)	TAG(54:2)	18:0/18:1/18:1	LC-MS Lipidomics	HMDB05403		↑
L86	Triacylglycerol	TAG(54:3)	TAG(54:3)	18:1/18:1/18:1	LC-MS Lipidomics	HMDB0005453		↑
L87	Triacylglycerol	TAG(54:4)	TAG(54:4)	8:2/18:1 or 18:2/18:	LC-MS Lipidomics			↑
L88	Triacylglycerol	TAG(54:5)	TAG(54:5)	8:1/18:2 or 18:1/18:	LC-MS Lipidomics			↑
L89	Triacylglycerol	TAG(54:6)	TAG(54:6)	8:1/18:3 or 18:2/16:	LC-MS Lipidomics			↑
L90	Triacylglycerol	TAG(54:7)	TAG(54:7)	8:2/18:3 or 18:2/18:	LC-MS Lipidomics			↑
L91	Triacylglycerol	TAG(54:8)	TAG(54:8)		LC-MS Lipidomics			↑
L92	Triacylglycerol	TAG(55:3)	TAG(55:3)		LC-MS Lipidomics			↑
L93	Triacylglycerol	TAG(56:0)	TAG(56:0)		LC-MS Lipidomics			↑
L94	Triacylglycerol	TAG(56:1)	TAG(56:1)	2:0/18:1 or 18:1/20:	LC-MS Lipidomics			↑
L95	Triacylglycerol	TAG(56:2)	TAG(56:2)	0:1/18:0 or 18:1/20:	LC-MS Lipidomics			↑
L96	Triacylglycerol	TAG(56:4)	TAG(56:4)		LC-MS Lipidomics			↑
L97	Triacylglycerol	TAG(56:5)	TAG(56:5)	or 18:1/20:3/18:1 or	LC-MS Lipidomics			↑
L98	Triacylglycerol	TAG(56:6)	TAG(56:6)	2:5/18:1 or 18:1/20:	LC-MS Lipidomics			↑
L99	Triacylglycerol	TAG(56:7)	TAG(56:7)	22:6/16:0/18:1	LC-MS Lipidomics			↑
L100	Triacylglycerol	TAG(56:8)	TAG(56:8)	8:2/16:0 and 18:2/18	LC-MS Lipidomics			↑
L101	Triacylglycerol	TAG(57:6)	TAG(57:6)		LC-MS Lipidomics			↓
L102	Triacylglycerol	TAG(58:10)	TAG(58:10)		LC-MS Lipidomics			↑
L103	Triacylglycerol	TAG(58:2)	TAG(58:2)	2:0/18:1 or 18:1/16:	LC-MS Lipidomics			↑
L104	Triacylglycerol	TAG(58:4)	TAG(58:4)		LC-MS Lipidomics			↑
L105	Triacylglycerol	TAG(58:6)	TAG(58:6)		LC-MS Lipidomics			↑
L106	Triacylglycerol	TAG(58:8)	TAG(58:8)		LC-MS Lipidomics			↑
L107	Triacylglycerol	TAG(60:2)	TAG(60:2)		LC-MS Lipidomics			↑
L108	Triacylglycerol	TAG(60:3)	TAG(60:3)		LC-MS Lipidomics			↑
L109	Triacylglycerol	TAG(60:4)	TAG(60:4)		LC-MS Lipidomics			↑
L110	Cardiolipin	CL(72:6)	CL(72:6)		LC-MS Lipidomics			↑
L111	Cardiolipin	CL(70:7)	CL(70:7)		LC-MS Lipidomics			↑
L112	Cardiolipin	CL(72:7)	CL(72:7)		LC-MS Lipidomics			↑
L113	Cardiolipin	CL(72:8)	CL(72:8)		LC-MS Lipidomics			↑
L114	Cardiolipin	CL(74:11)	CL(74:11)		LC-MS Lipidomics			↑
R1	PC:PE	LPC:LPE (16:0)	LPC:LPE (16:0)		LC-MS Metabolomics			↓
R2	PC:PE	LPC:LPE (18:0)	LPC:LPE (18:0)		LC-MS Metabolomics			↓
R3	PC:PE	LPC:LPE (18:1)	LPC:LPE (18:1)		LC-MS Metabolomics			↓
R4	PC:PE	LPC:LPE (18:2)	LPC:LPE (18:2)		LC-MS Metabolomics			↓
R5	PC:PE	PC/PE(34:1)	PC:PE(34:1)		Lipidomics			↑
R6	PC:PE	PC/PE(36:1)	PC:PE(36:1)		Lipidomics			↑
R7	PC:PE	PC/PE(34:2)	PC:PE(34:2)		Lipidomics			↑
R8	PC:PE	PC/PE(36:2)	PC:PE(36:2)		Lipidomics			↓
R9	PC:PE	PC/PE(36:4)	PC:PE(36:4)		Lipidomics			↓
R10	PC:PE	PC/PE(38:4)	PC:PE(38:4)		Lipidomics			↓
R11	PC:PE	PC/PE(38:5)	PC:PE(38:5)		Lipidomics			↓
R12	PC:PE	PC/PE(40:5)	PC:PE(40:5)		Lipidomics			↓
L129	Other lipid	PS(40:6)	PS(40:6)	PS(18:0/22:6)	LC-MS Lipidomics	HMDB0010167		↑
L140	Other lipid	PG(34:0)	PG(34:0)		LC-MS Lipidomics			↑
G150	Other lipid	1-Monopalmitoylglycerol	1-MPG		GC-MS Metabolomics	HMDB31074	542-44-9	↓
G151	Other lipid	1-Monostearoylglycerol	1-MSG		GC-MS Metabolomics			↑
G152	Other lipid	Cholesterol	Chol		GC-MS Metabolomics	HMDB00067		↓

G153	Other lipid	Glycerol-2-	G2	GC-MS	HMDB00126		↓
G154	Other lipid	phosphate	P	Metabolomics GC-	HMDB001347		↓
G155	Other	Glycerol-3-	G3	MS Metabolomics	8 HMDB00256		↑
L180	lipid	phosphate	P	GC-MS	HMDB00086		↓
	Other lipid	Squalene	Squ	Metabolomics			
		Glycerophosphocholine	GPC	LC-MS Metabolomics			
L253	Other lipid	CMPF	CMPF	LC-MS Metabolomics			↓
L163	Acylcarnitine	L-Carnitine	L-Carnitine	LC-MS Metabolomics	HMDB00062		↓
L168	Acylcarnitine	Acetyl-carnitine (C2)	C2-carn	LC-MS Metabolomics	HMDB00201	3040-38-8	↓
L171	Acylcarnitine	Propionyl-carnitine (C3)	C3-carn	LC-MS Metabolomics	HMDB00824	17298-37-2	↓
L174	Acylcarnitine	Butyryl-carnitine (C4)	C4-carn	LC-MS Metabolomics	HMDB02013	25576-40-3	↓
L176	Acylcarnitine	Tiglyl-carnitine (C5:1iso)	C5:1-iso-carn	LC-MS Metabolomics	HMDB02366	64191-86-2	↓
L177	Acylcarnitine	Valeryl-carnitine (C5)	C5-carn	LC-MS Metabolomics	HMDB13128		↓
L178	Acylcarnitine	Isovaleryl-carnitine (C5iso)	C5iso-carn	LC-MS Metabolomics	HMDB00688	31023-24-2	↓
L181	Acylcarnitine	Hexanoyl-carnitine (C6)	C6-carn	LC-MS Metabolomics	HMDB00705	6418-78-6	↓
L182	Acylcarnitine	Hydroxyisovaleryl-carnitine (C5iso-OH)	C5iso-OH-carn	LC-MS Metabolomics	HMDB61189		↓
L184	Acylcarnitine	Glutaroyl-carnitine (C5-DC)	C5-DC-carn	LC-MS Metabolomics	HMDB62587	102636-82-8	↑
L185	Acylcarnitine	Octenoyl-carnitine (C8:1)	C8:1-carn	LC-MS Metabolomics	HMDB13324		↓
L186	Acylcarnitine	Octanoyl-carnitine (C8)	C8-carn	LC-MS Metabolomics	HMDB00791	25243-95-2	↓
L189	Acylcarnitine	Decenoyl-carnitine (C10:1)	C10:1-carn	LC-MS Metabolomics			↓
L190	Acylcarnitine	Decanoyl-carnitine (C10:0)	C10:0-carn	LC-MS Metabolomics	HMDB00651		↓
L191	Acylcarnitine	Hydroxydecanoyl-carnitine (C10:0-OH)	C10-OH-carn	LC-MS Metabolomics			↓
L192	Acylcarnitine	Dodecenoyl-carnitine (C12:1)	C12:1-carn	LC-MS Metabolomics	HMDB13326		↓
L193	Acylcarnitine	Dodecanoyl-carnitine (C12:0)	C12-carn	LC-MS Metabolomics	HMDB02250	25518-54-1	↓
L195	Acylcarnitine	Hydroxylauroyl-carnitine (C12:0-OH)	C12-OH-carn	LC-MS Metabolomics			↓
L196	Acylcarnitine	Tetradecdienoyl-carnitine (C14:2)	C14:2-carn	LC-MS Metabolomics			↓
L197	Acylcarnitine	Tetradecenoyl-carnitine (C14:1)	C14:1-carn	LC-MS Metabolomics		835598-21-5	↓
L198	Acylcarnitine	Myristoyl-carnitine (C14:0)	C14-carn	LC-MS Metabolomics	HMDB05066	25597-07-3	↓
L199	Acylcarnitine	Hydroxymyristoyl-carnitine (C14:0-OH)	C14:0-OH-carn	LC-MS Metabolomics			↓
L200	Acylcarnitine	Hexadecdienoyl carnitine (C16:2)	C16:2-carn	LC-MS Metabolomics			↓
L201	Acylcarnitine	Hexadecenoyl-carnitine (C16:1)	C16:1-carn	LC-MS Metabolomics			↓
L202	Acylcarnitine	Palmitoyl-carnitine (C16:0)	C16-carn	LC-MS Metabolomics	HMDB00222	2364-67-2	↓
L203	Acylcarnitine	Hydroxypalmitoleoyl-carnitine (C16:1-O	C16:1-OH-carn	LC-MS Metabolomics			↓
L204	Acylcarnitine	Linoleneoyl-carnitine (C18:3)	C18:3-carn	LC-MS Metabolomics	HMDB06319		↑
L205	Acylcarnitine	Linoleoyl-carnitine (C18:2)	C18:2-carn	LC-MS Metabolomics	HMDB06469	36816-10-1	↑
L206	Acylcarnitine	Oleoyl-carnitine (C18:1)	C18:1-carn	LC-MS Metabolomics		38677-66-6	↑
L207	Acylcarnitine	Stearoyl-carnitine (C18:0)	C18-carn	LC-MS Metabolomics	HMDB00848	1976-27-8	↑
L208	Acylcarnitine	Hydroxylinoleoyl-carnitine (C18:2-OH)	C18:2-OH-carn	LC-MS Metabolomics			↓
L209	Acylcarnitine	Eicosadieneoyl-carnitine (C20:2)	C20:2-carn	LC-MS Metabolomics			↑
L210	Acylcarnitine	Eicoseneoyl-carnitine (C20:1)	C20:1-carn	LC-MS Metabolomics			↓
L115	Acylcarnitine	Decanoyl-carnitine (C10:0)	C10:0-carn	LC-MS Lipidomics	HMDB62631		↓
L116	Acylcarnitine	Dodecanoyl-carnitine (C12:0)	C12:0-carn	LC-MS Lipidomics	HMDB02250	25518-54-1	↑
L117	Acylcarnitine	Myristoyl-carnitine (C14:0)	C14:0-carn	LC-MS Lipidomics	HMDB05066	25597-07-3	↑
L118	Acylcarnitine	Palmitoyl-carnitine (C16:0)	C16:0-carn	LC-MS Lipidomics		2364-67-2	↑
L119	Acylcarnitine	Stearoyl-carnitine (C18:0)	C18:0-carn	LC-MS Lipidomics	HMDB06463	1976-27-8	↑
L120	Acylcarnitine	Decenoyl-carnitine (C10:1)	C10:1-carn	LC-MS Lipidomics			↑
L121	Acylcarnitine	Dodecenoyl-carnitine (C12:1)	C12:1-carn	LC-MS Lipidomics	HMDB13326		↑
L122	Acylcarnitine	Tetradecenoyl-carnitine (C14:1)	C14:1-carn	LC-MS Lipidomics		835598-21-5	↑
L123	Acylcarnitine	Hexadecenoyl-carnitine (C16:1)	C16:1-carn	LC-MS Lipidomics			↑
L124	Acylcarnitine	Oleoyl-carnitine (C18:1)	C18:1-carn	LC-MS Lipidomics		38677-66-6	↑
L125	Acylcarnitine	Hexadecdienoyl-carnitine (C16:2)	C16:2-carn	LC-MS Lipidomics			↑
L126	Acylcarnitine	Linoleoyl-carnitine (C18:2)	C18:2-carn	LC-MS Lipidomics	HMDB06461	36816-10-1	↑
L127	Acylcarnitine	Eicosaenoyl-carnitine (20:3)	C20:3-carn	LC-MS Lipidomics			↑
L128	Acylcarnitine	Arachidonyl-carnitine(20:4)	C20:4-carn	LC-MS Lipidomics	HMDB06455		↑
G1	Amino acid	2-Amino-adipic acid	Aad	GC-MS Metabolomics			↓
G2	Amino acid	2-Aminobutyric acid	Abu	GC-MS Metabolomics	HMDB00452	1492-24-6	↓
G3	Amino acid	Alanine	Ala	GC-MS Metabolomics	HMDB00161	56-41-7	↑
G4	Amino acid	Allothreonine	allo-Thr	GC-MS Metabolomics	HMDB04041	24830-94-2	↑
G59	Amino acid	Aminomalonic Acid	Ama	GC-MS Metabolomics	HMDB01147		↓
G5	Amino acid	Arginine	Arg	GC-MS Metabolomics	HMDB00517	74-79-3	↓
G6	Amino acid	Asparagine	Asp	GC-MS Metabolomics	HMDB00168	70-47-3	↓
G7	Amino acid	Asymetrical-n,n-dimethylarginine	ADMA	GC-MS Metabolomics	HMDB01539	30315-93-6	↓
L173	Amino acid	Carnosine		LC-MS Metabolomics	HMDB00033		↑
G10	Amino acid	Citrulline(arginine)	Cit-a	GC-MS Metabolomics	HMDB00904	372-75-8	↓
G9	Amino acid	Citrulline(ornthine)	Cit-o	GC-MS Metabolomics	HMDB00904	372-75-8	↓
L156	Amino acid	Creatine	Cr	LC-MS Metabolomics	HMDB00064		↓
G11	Amino acid	Creatinine	Cre	GC-MS Metabolomics	HMDB00562	60-27-5	↑
L152	Amino acid	Creatinine	Cre	LC-MS Metabolomics	HMDB00562	60-27-5	↓
L165	Amino acid	DL-Tyrosine	Tyr	LC-MS Metabolomics	HMDB00158		↑
L247	Amino acid	DL-Tyrosine	Tyr	LC-MS Metabolomics	HMDB00158		↑
G13	Amino acid	Glutamic acid	Glu	GC-MS Metabolomics	HMDB03339	6893-26-1	↓
G14	Amino acid	Glutamine	Gln	GC-MS Metabolomics	HMDB00641		↓
L188	Amino acid	Glutathione (GSH) reduced		LC-MS Metabolomics	HMDB00641	56-85-9	↓
L223	Amino acid	Glutathione oxidized		LC-MS Metabolomics	HMDB03337		↓
G17	Amino acid	Glycine	Gly	GC-MS Metabolomics	HMDB00123	56-40-6	↓
G19	Amino acid	Glycylvaline	Gly-val	GC-MS Metabolomics	HMDB28854	1963-21-9	↑
L246	Amino acid	Hippuric acid	HA	LC-MS Metabolomics	HMDB00714	495-69-2	↑
G39	Amino acid	Histidine	His	GC-MS Metabolomics	HMDB00177	71-00-1	↓
G20	Amino acid	Isoleucine	Ile	GC-MS Metabolomics	HMDB00172	73-32-5	↑
G36	Amino acid	Leucine	Leu	GC-MS Metabolomics	HMDB00687	61-90-5	↑
L157	Amino acid	L-Isoleucine	Ile	LC-MS Metabolomics	HMDB00172	73-32-5	↑
L169	Amino acid	L-Kynurenine	Kyn	LC-MS Metabolomics	HMDB00684	343-65-7	↑
L158	Amino acid	L-Leucine	Leu	LC-MS Metabolomics	HMDB00687	61-90-5	↑
L240	Amino acid	L-Leucine	Leu	LC-MS Metabolomics	HMDB00687	61-90-5	↑
L161	Amino acid	L-Methionine	Met	LC-MS Metabolomics	HMDB00696	63-68-3	↑
L153	Amino acid	L-Proline	Pro	LC-MS Metabolomics	HMDB00162	147-85-3	↓
L167	Amino acid	L-Tryptophan	Trp	LC-MS Metabolomics	HMDB00929	73-22-3	↑
L250	Amino acid	L-Tryptophan	Trp	LC-MS Metabolomics	HMDB00929	73-22-3	↑
G22	Amino acid	Lysine	Lys	GC-MS Metabolomics	HMDB00182	56-87-1	↑
G23	Amino acid	N-acetylornthine	OrnNAc	GC-MS Metabolomics			↓
L187	Amino acid	Ophthalmic acid		LC-MS Metabolomics	HMDB05765		↓
G24	Amino acid	Ornithine	Orn	GC-MS Metabolomics	HMDB00214	70-26-8	↓
G26	Amino acid	Phenylalanine	Phe	GC-MS Metabolomics	HMDB00159	63-91-2	↑
L164	Amino acid	Phenylalanine	Phe	LC-MS Metabolomics	HMDB00159	63-91-2	↑
L244	Amino acid	Phenylalanine	Phe	LC-MS Metabolomics	HMDB00159	63-91-2	↑
L170	Amino acid	Phosphocreatine		LC-MS Metabolomics	HMDB01511		↓
G27	Amino acid	Pipecolic acid	PipA	GC-MS Metabolomics	HMDB00070	535-75-1	↓
G28	Amino acid	Proline	Pro	GC-MS Metabolomics	HMDB00162	147-85-3	↓
G29	Amino acid	Pyroglutamic acid	PyrA	GC-MS Metabolomics	HMDB00267	98-79-3	↓
L155	Amino acid	Pyroglutamic acid	PyrA	LC-MS Metabolomics	HMDB00267	98-79-3	↓
L238	Amino acid	Pyroglutamic acid	PyrA	LC-MS Metabolomics	HMDB00267	98-79-3	↓
G30	Amino acid	Sarcosine	Sar	GC-MS Metabolomics	HMDB00271	107-97-1	↑

G31	Amino acid	Serine	Ser	GC-MS Metabolomics	HMDB00187		↓
G32	Amino acid	Taurine	Tau	GC-MS Metabolomics	HMDB00251	107-35-7	↑
G33	Amino acid	Threonine	Thr	GC-MS Metabolomics	HMDB00167	72-19-5	↑
G34	Amino acid	Tyrosine	Tyr	GC-MS Metabolomics	HMDB00158	60-18-4	↑
G35	Amino acid	Valine	Val	GC-MS Metabolomics	HMDB00883	72-18-4	↑
G8	Amino acid	β-alanine	β-Ala	GC-MS Metabolomics	HMDB00056	107-95-9	↓
G12	Amino acid	γ-glutamylleucine	GLN-Leu	GC-MS Metabolomics		2566-39-4	↑
G183	Carboxylic acid	2-Hydroxy-3-Methylbutyric Acid	2-HIV	GC-MS Metabolomics	HMDB00407		↑
G159	Carboxylic acid	2-Methylmalic acid	CitMA	GC-MS Metabolomics	HMDB00426	597-44-4	↑
G173	Carboxylic acid	3-Hydroxybutyric acid	3-HB	GC-MS Metabolomics	HMDB00357	300-85-6	↓
G172	Carboxylic acid	3-Hydroxyisobutyric acid	3-HIB	GC-MS Metabolomics	HMDB00023	2068-83-9	↓
G160	Carboxylic acid	4-Hydroxybenzoic acid	PHBA	GC-MS Metabolomics	HMDB00500		↓
G162	Carboxylic acid	Azelaic acid	AZA	GC-MS Metabolomics	HMDB00784	123-99-9	↓
G163	Carboxylic acid	Citric acid	CitA	GC-MS Metabolomics	HMDB00094	77-92-9	↑
L248	Carboxylic acid	Citric acid	CitA	LC-MS Metabolomics	HMDB00094	77-92-9	↑
G164	Carboxylic acid	Fumaric acid	FA	GC-MS Metabolomics	HMDB00134	110-17-8	↑
G175	Carboxylic acid	Galacturonic acid	GalA	GC-MS Metabolomics	HMDB02545		↑
G176	Carboxylic acid	Glutaric acid	GluA	GC-MS Metabolomics	HMDB00661	110-94-1	↑
G177	Carboxylic acid	Glycolic Acid	GlyA	GC-MS Metabolomics	HMDB00115	79-14-1	↓
G165	Carboxylic acid	Isocitric acid	Iso-CitA	GC-MS Metabolomics	HMDB00193	320-77-4	↑
L239	Carboxylic acid	Ketoleucine	K-leu	LC-MS Metabolomics	HMDB00695	816-66-0	↑
G166	Carboxylic acid	Lactic acid	LacA	GC-MS Metabolomics	HMDB00190	79-33-4	↑
L237	Carboxylic acid	L-Lactic acid	LacA	LC-MS Metabolomics	HMDB00190	79-33-4	↑
G167	Carboxylic acid	Maleic acid	MalA	GC-MS Metabolomics	HMDB00176		↑
G168	Carboxylic acid	Malic acid	MA	GC-MS Metabolomics	HMDB00744	6915-15-7	↑
L241	Carboxylic acid	Malic acid	MA	LC-MS Metabolomics	HMDB00744	6915-15-7	↑
G169	Carboxylic acid	Oxalic acid	OA	GC-MS Metabolomics	HMDB02329	144-62-7	↑
G170	Carboxylic acid	Oxaloacetic acid	K-Suc	GC-MS Metabolomics			↓
G174	Carboxylic acid	Pyruvic acid	PyruA	GC-MS Metabolomics	HMDB00243	127-17-3	↑
G171	Carboxylic acid	Succinic acid	SucA	GC-MS Metabolomics	HMDB00254	110-15-6	↓
G161	Carboxylic acid	α-ketoglutaric acid	α-KG	GC-MS Metabolomics	HMDB00208	328-50-7	↑
G64	Carbohydrate	1,5-anhydro-D-Glucitol	1,5-AG	GC-MS Metabolomics	HMDB02712	154-58-5	↑
G65	Carbohydrate	2-Deoxy-glucose	2DG	GC-MS Metabolomics	HMDB12327	25494-04-6	↓
G90	Carbohydrate	allo-Inositol	a-Ino-ol	GC-MS Metabolomics			↑
G132	Carbohydrate	Alpha-Lactose	α-Lac	GC-MS Metabolomics	HMDB00186		↑
G66	Carbohydrate	Cellobiose	Glc2	GC-MS Metabolomics	HMDB00055		↓
G68	Carbohydrate	Cellotriose	Glc3	GC-MS Metabolomics			↓
G91	Carbohydrate	chiro-Inositol	c-Ino-ol	GC-MS Metabolomics			↑
G69	Carbohydrate	Erythritol	Ery-ol	GC-MS Metabolomics	HMDB02994		↑
G71	Carbohydrate	Erythrose	Ery	GC-MS Metabolomics	HMDB02649		↑
G70	Carbohydrate	Erythrose-4-phosphate	E4P	GC-MS Metabolomics	HMDB01321		↓
G76	Carbohydrate	Fructose	Fru	GC-MS Metabolomics	HMDB00660	53188-23-1	↑
G72	Carbohydrate	Fructose-1,6-disphosphate	FDP	GC-MS Metabolomics	HMDB01058		↓
G73	Carbohydrate	Fructose-1-phosphate	F1P	GC-MS Metabolomics	HMDB01076		↑
G75	Carbohydrate	Fructose-6-phosphate	F6P	GC-MS Metabolomics	HMDB00124		↑
G77	Carbohydrate	Galactitol	Gal-ol	GC-MS Metabolomics	HMDB00107	608-66-2	↓
G78	Carbohydrate	Galactose	Gal	GC-MS Metabolomics	HMDB00143	59-23-4	↓
G85	Carbohydrate	Glucose	Glc	GC-MS Metabolomics	HMDB00122	50-99-7	↓
G83	Carbohydrate	Glucose-6-phosphate	G6P	GC-MS Metabolomics	HMDB01401		↑
G87	Carbohydrate	Glucuronic acid	GlcA	GC-MS Metabolomics	HMDB00127		↓
G88	Carbohydrate	Glyceric acid-2-phosphate	2PGA	GC-MS Metabolomics			↓
G89	Carbohydrate	Glyceric acid-3-phosphate	3PGA	GC-MS Metabolomics			↓
G92	Carbohydrate	myo-Inositol	m-Ino-ol	GC-MS Metabolomics	HMDB00211	87-89-8	↑
G94	Carbohydrate	Isomaltose	Iso-mat	GC-MS Metabolomics	HMDB02923		↓
G95	Carbohydrate	Lactitol	Lac-ol	GC-MS Metabolomics			↑
G96	Carbohydrate	Lactose	Lac	GC-MS Metabolomics	HMDB41627	5965-66-2	↑
G97	Carbohydrate	Lactulose	Lactu	GC-MS Metabolomics	HMDB00740	4618-18-2	↓
G98	Carbohydrate	Laminaribiose	Lam	GC-MS Metabolomics		34980-39-7	↓
G99	Carbohydrate	Maltitol	Mal-ol	GC-MS Metabolomics	HMDB02928		↑
G100	Carbohydrate	Maltose	Mal	GC-MS Metabolomics	HMDB00163	69-79-4	↑
G101	Carbohydrate	Maltotriose	Malto	GC-MS Metabolomics	HMDB01262		↑
G102	Carbohydrate	Mannitol	Man-ol	GC-MS Metabolomics	HMDB00765	69-65-8	↑
G103	Carbohydrate	Mannose	Man	GC-MS Metabolomics	HMDB00169	3458-28-4	↓
G106	Carbohydrate	Melibiose	Mel	GC-MS Metabolomics	HMDB00048		↓
G107	Carbohydrate	N-acetyl glucosamine	GluNAc	GC-MS Metabolomics	HMDB00215	7512-17-6	↑
G108	Carbohydrate	N-acetyl mannosamine	ManNAc	GC-MS Metabolomics	HMDB01129	3615-17-6	↓
G109	Carbohydrate	Palatinose	Pal	GC-MS Metabolomics			↓
G120	Carbohydrate	Ribose-5-phosphate	Rib5P	GC-MS Metabolomics	HMDB01548		↑
G110	Carbohydrate	Sakebiose	Sak	GC-MS Metabolomics	HMDB29882	497-48-3	↓
G93	Carbohydrate	scyllo-Inositol	s-Ino-ol	GC-MS Metabolomics	HMDB06088	488-59-5	↑
G113	Carbohydrate	Sorbitol	Sor-ol	GC-MS Metabolomics	HMDB05831	20479-58-7	↑
G111	Carbohydrate	Sorbitol-6-phosphate	Sor6P	GC-MS Metabolomics	HMDB05831	20479-58-7	↑
G114	Carbohydrate	Sorbose	Sor	GC-MS Metabolomics	HMDB01266	87-79-6	↑
G117	Carbohydrate	Sucrose	Suc	GC-MS Metabolomics	HMDB00258	57-50-1	↑
G118	Carbohydrate	Trehalose	Tre	GC-MS Metabolomics	HMDB00975	99-20-7	↑
G119	Carbohydrate	Xylitol	Xyl-ol	GC-MS Metabolomics	HMDB02917	87-99-0	↑
L254	No class	2- Hydroxy-3-(4-methoxyethylphenoxy)-propanoic		LC-MS			↓
L179	No class	acid 3-Hydroxylidocaine		Metabolomics LC-			↓
G210	No class	3- Pyridinol	3-Pyr	MS Metabolomics			↓
L263	No class	3-β-hydroxyandrost-5-en-17-one sulfate		GC-MS	HMDB01032	651-48-9	↓
				Metabolomics			
				LC-MS Metabolomics			
G211	No class	5,6-Dihydrouracil	5,6-DHR	GC-MS Metabolomics	HMDB00076		↑
G184	No class	Acetaminophen	AAP	GC-MS Metabolomics	HMDB01859	103-90-2	↑
L160	No class	Acetylcholine	ACh	LC-MS Metabolomics	HMDB00895		↓
G186	No class	Adenine	Ade	GC-MS Metabolomics	HMDB00034		↓
L183	No class	Adenosine	Ade	LC-MS Metabolomics	HMDB00050	58-61-7	↓
G185	No class	Adenosine-5-monophosphate	AMP	GC-MS	HMDB00045	61-19-8	↓
G188	No class	Amiloride	Am	Metabolomics GC-			↑
G215	No class	Butylated Hydroxytoluene	BHT	MS Metabolomics			↑
L166	No class	Caffeine	Caffeine	GC-MS	HMDB0184		↑
L270	No class	Cyclic adenosine diphosphate ribose	CADR	Metabolomics LC-			↑
G189	No class	Ethanolamine	ETA	MS Metabolomics	7		↑
G191	No class	Gibberellin A20	GA20	LC-MS			↑
G192	No class	Gluconic acid 1,4-lactone	GGL	Metabolomics GC-	HMDB0014		↑
G193	No class	Gluconic acid 1,5-lactone	GDL	MS Metabolomics			↓
G194	No class	Glucuronic acid-E-lactone	GNL	GC-MS	9		↓
G203	No class	Hypoxanthine	Hyp	Metabolomics GC-		68-94-0	↑
				MS Metabolomics			
				GC-MS			
				Metabolomics GC-			
				MS Metabolomics	HMDB00157		
				GC-MS Metabolomics			
L159	No class	Hypoxanthine	Hyp	LC-MS Metabolomics	HMDB00157	68-94-0	↓
L242	No class	Hypoxanthine	Hyp	LC-MS Metabolomics	HMDB00157	68-94-0	↑
G195	No class	Inosine	Ino	GC-MS Metabolomics	HMDB00195	58-63-9	↓
L257	No class	Inosine	Ino	LC-MS Metabolomics	HMDB00195	58-63-9	↓

L194	No class	Inosine 5'-	IMP	LC-MS			↓
G19	No class	monophosphate	Lid	Metabolomics GC-			↑
6	No class	Lidocaine		MS Metabolomics			↓
L175		Lidocaine		LC-MS Metabolomics			
L154	No class	Niacinamide	VB3	LC-MS Metabolomics	HMDB01406	98-92-0	↑
G197	No class	Nicotinamide	VB3	GC-MS Metabolomics			↓
G198	No class	O-phosphoetanolamine	O-PE	GC-MS Metabolomics			↑
G213	No class	Ornithine-1,5-Lactam	Orn-1,5-L	GC-MS Metabolomics			↓
L162	No class	p-Acetamidophenol		LC-MS Metabolomics			↓
G199	No class	Pantothenic acid	PanA	GC-MS Metabolomics	HMDB00210		↑
L172	No class	Pantothenic acid	PanA	LC-MS Metabolomics	HMDB00210		↑
L251	No class	Pantothenic acid	PanA	LC-MS Metabolomics	HMDB00210		↑
G209	No class	Phosphate-Fragment	P	GC-MS Metabolomics			↓
G216	No class	Phthalic Acid, Di(2-Propylpentyl) Ester	PADE	GC-MS Metabolomics			↓
G201	No class	Putrescine	Put	GC-MS Metabolomics	HMDB01414		↓
G214	No class	Pyrophosphate	PPi	GC-MS Metabolomics	HMDB00250		↓
L264	No class	S-Adenosylhomocysteine	AdoHcy	LC-MS Metabolomics	HMDB00939		↑
G204	No class	Urea	Urea	GC-MS Metabolomics	HMDB00294		↓
G202	No class	Uric acid	UA	GC-MS Metabolomics	HMDB00289		↑
L245	No class	Uric acid	UA	LC-MS Metabolomics			↓
G187	No class	α-tocopherol	α-VE	GC-MS Metabolomics	HMDB01893		↑
G190	No class	γ-tocopherol	γ-VE	GC-MS Metabolomics	HMDB01492		↑
G158	No class	Inositol-Phosphate	IP	GC-MS Metabolomics	HMDB00213		↓

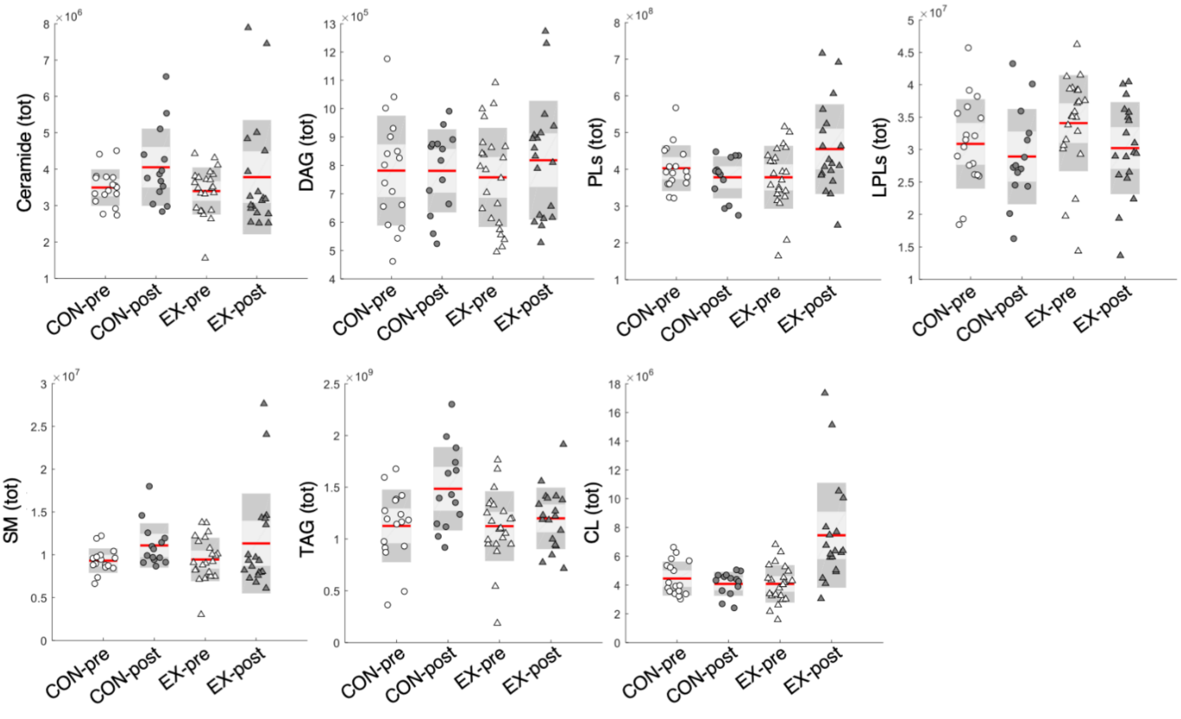

ESM Fig. 1. Total content of lipid subtypes in skeletal muscle at the different time points in control (CON) and exercise (EX) groups. The mean relative abundance of ceramides and diacylglycerols (DAGs) is around 10^5, phospholipids (PLs) 10^8, lysophospholipids (LPL) and sphingomyelins 10^7, whereas triacylglycerols (TAGs) are 10^9 and cardiolipins (CL) around 10^6. Raw data for unique lipid species are found in the supplementary Table 1.

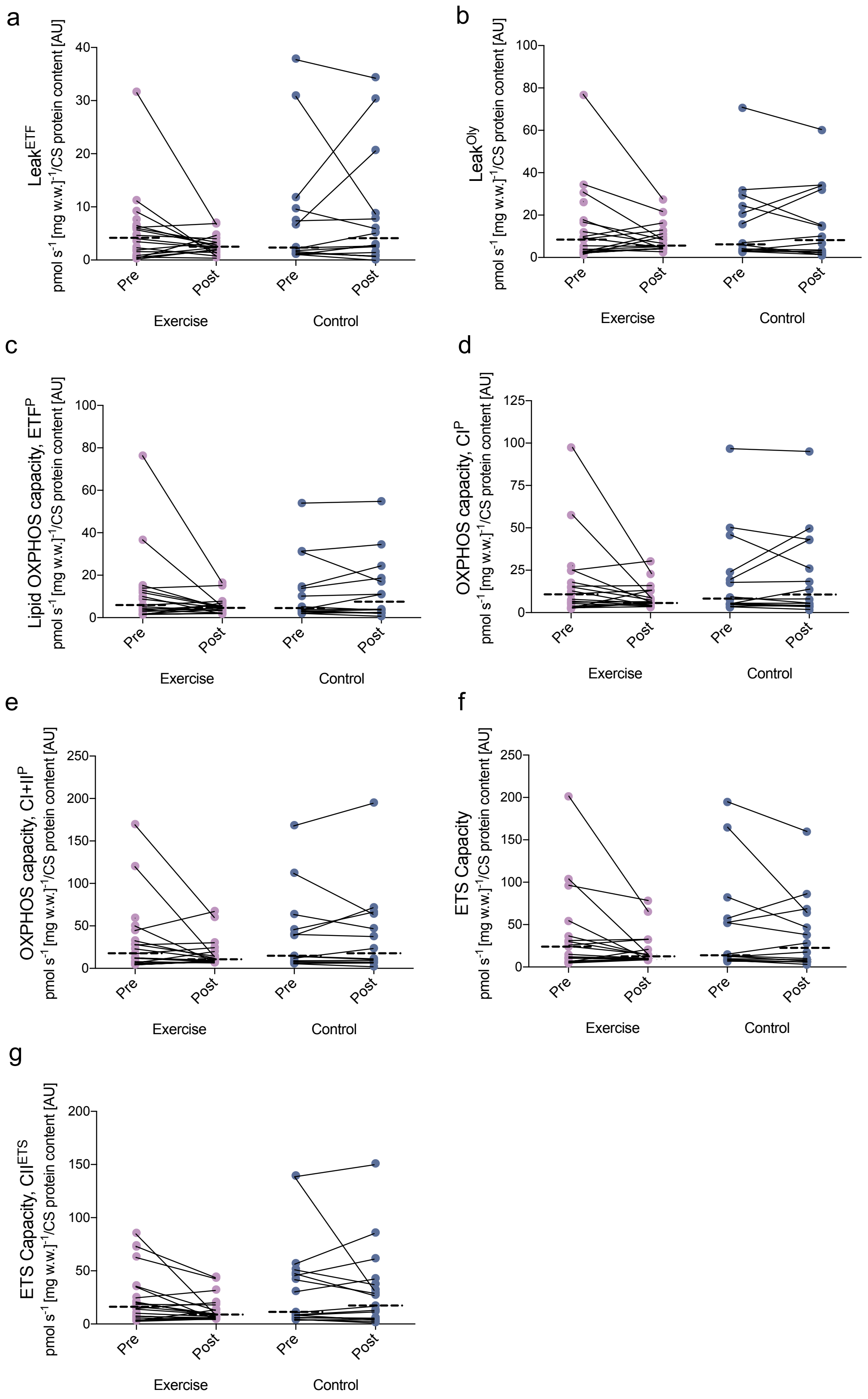

ESM Fig.2. Mitochondrial function across all respiratory states when adjusted for citrate synthase (CS) protein content as a marker of mitochondrial content (pmol s^{-1} [mg w.w.]$^{-1}$/CS protein content [AU]) in response to the 12-week intervention. Repeated measures ANOVA was used to identify main effects of time (pre and post), group (exercise and control), and interaction (group x time) in exercise (n=18) and control (n=14) groups. Dashed line represents median.

References

[1] Pesta D, Gnaiger E (2012) High-resolution respirometry: OXPHOS protocols for human cells and permeabilized fibers from small biopsies of human muscle. In: Mitochondrial Bioenergetics. Springer, pp 25-58

[2] Jacobs RA, Flück D, Bonne TC, et al. (2013) Improvements in exercise performance with high-intensity interval training coincide with an increase in skeletal muscle mitochondrial content and function. J Appl Physiol 115(6): 785-793

[3] Larsen S, Danielsen JH, Søndergård SD, et al. (2015) The effect of high-intensity training on mitochondrial fat oxidation in skeletal muscle and subcutaneous adipose tissue. Scand J Med Sci Sports 25(1): e59-e69

[4] Jonsson P, Wuolikainen A, Thysell E, et al. (2015) Constrained randomization and multivariate effect projections improve information extraction and biomarker pattern discovery in metabolomics studies involving dependent samples. Metabolomics 11(6): 1667-1678

[5] Chorell E, Ryberg M, Larsson C, et al. (2016) Plasma metabolomic response to postmenopausal weight loss induced by different diets. Metabolomics 12(5). ARTN 85 10.1007/s11306-016-1013-x
